# Mammalian type opsin 5 preferentially activates G14 in Gq-type G proteins triggering intracellular calcium response

**DOI:** 10.1016/j.jbc.2023.105020

**Published:** 2023-07-07

**Authors:** Keita Sato, Takahiro Yamashita, Hideyo Ohuchi

**Affiliations:** 1 Department of Cytology and Histology, Faculty of Medicine, Dentistry, and Pharmaceutical Sciences, Okayama University, Okayama City, Okayama, Japan; 2 Department of Biophysics, Graduate School of Science, Kyoto University, Kyoto, Japan

**Keywords:** G protein, G protein−coupled receptor (GPCR), photoreceptor, rhodopsin, calcium intracellular release, protein−protein interaction, signal transduction, nonvisual photoreception

## Abstract

Mammalian type opsin 5 (Opn5m), a UV-sensitive G protein–coupled receptor opsin highly conserved in vertebrates, would provide a common basis for UV sensing from lamprey to humans. However, G protein coupled with Opn5m remains controversial due to variations in assay conditions and the origin of Opn5m across different reports. Here, we examined Opn5m from diverse species using an aequorin luminescence assay and Gα-KO cell line. Beyond the commonly studied major Gα classes, Gαq, Gα11, Gα14, and Gα15 in the Gq class were individually investigated in this study, as they can drive distinct signaling pathways in addition to a canonical calcium response. UV light triggered a calcium response *via* all the tested Opn5m proteins in 293T cells, which was abolished by Gq-type Gα deletion and rescued by cotransfection with mouse and medaka Gq-type Gα proteins. Opn5m preferentially activated Gα14 and close relatives. Mutational analysis implicated specific regions, including α3-β5 and αG-α4 loops, αG and α4 helices, and the extreme C terminus, in the preferential activation of Gα14 by Opn5m. FISH revealed co-expression of genes encoding Opn5m and Gα14 in the scleral cartilage of medaka and chicken eyes, supporting their physiological coupling. This suggests that the preferential activation of Gα14 by Opn5m is relevant for UV sensing in specific cell types.

The function of G protein–coupled receptors (GPCRs) is primarily mediated by heterotrimeric G proteins (1, 2). Heterotrimeric G proteins are composed of α, β, and γ subunits (3). Among them, the α subunit has a guanosine diphosphate/guanosine triphosphate (GTP)-binding domain and primarily determines coupling specificity with the receptors. There are five major classes of α subunits, Gi, Gq, Gs, G12, and Gv in vertebrates (4). Typically, members of the Gi and Gs classes decrease or increase intracellular cyclic adenosine monophosphate (cAMP) levels by inhibiting or enhancing the activity of adenylate cyclase, respectively (5). Members of the Gq class activate phospholipase C (PLC) to degrade phosphatidylinositol 4,5-diphosphate to diacylglycerol (DAG) and inositol triphosphate (IP3) (6). Accordingly, DAG activates the protein kinase C, and IP3 opens the IP3 receptor, a calcium channel located at the endoplasmic reticulum membrane. Members of the G12 class function by activating the Rho family small G protein (7). The Gv class is lost in tetrapods, and its biochemical properties have not been well investigated (8).

Each of the major classes of α subunits consists of multiple member proteins. In the Gq class, there are four members: Gαq, Gα11, Gα14, and Gα15 (9). While these members share a common canonical pathway for activating PLC, there is evidence showing the different roles and signaling functions including the sensitization of nociception, interaction with tetratricopeptide repeat 1, activation of NFκB, and activation of c-Jun N-terminal kinase (10, 11, 12, 13, 14, 15). Activation of Gαq, Gα14, and Gα15 in vascular smooth muscle cells induce upregulations of respective different gene sets (16). These examples show that Gq class members, Gαq, Gα11, Gα14, and Gα15, are not just redundant signal transducers and that precise investigation of receptor-G coupling considering these subclasses, and not only major Gi/s/q/12/v, contributes to understanding the molecular physiology of GPCRs.

Opsin is an animal photoreceptor protein that uses retinylidene imine covalently bound to the conserved lysine residue as a chromophore (17). This is also a member of the GPCR family. One of the most well-studied animal opsins is vertebrate rhodopsin, the photoreceptor for scotopic image–forming vision. Upon light absorption, vertebrate rhodopsin activates transducin, a subclass of Gi-type G protein, leading to the activation of phosphodiesterase, a decrease of intracellular cyclic guanosine monophosphate (cGMP) concentration, closure of the cGMP-gated channel, depolarization of membrane potential, and arrest of glutamate release from the presynaptic terminal (18). In invertebrate species, visual opsins are Gq-coupled in arthropods and mollusks and Gs-coupled in box jellyfish. While the intracellular processes in the photoreceptor cells of these animals are essentially different from those in vertebrates, their functions are convergent to photoreception in image-forming vision (19, 20).

In addition to opsins involved in image-forming vision, there are multiple opsin gene homologs in the animal genome. Opsins that are thought to be primarily involved in non-image-forming photoreception are collectively called nonvisual or non-image-forming opsins. Opsin 5/neuropsin (Opn5) is a non-image-forming opsin that was discovered by Tarttelin *et al.* (21) from mice and humans in 2003. *Opn5* gene homologs are widely found in the genomes of nonmammalian vertebrates, tardigrades, brachiopods, annelids, etc. and are presumed to have been acquired by a common ancestor of bilaterians (22, 23). Among vertebrates, therian mammals have only one Opn5, while nonmammalian vertebrates and monotremes have multiple Opn5 homologs, which are classified into separate phylogenetic groups, mammalian type Opn5 (Opn5m), opsin 5-like 1 (Opn5L1), and opsin 5-like 2 (Opn5L2) (23). The ortholog of mammalian Opn5 is referred to as Opn5m. Additionally, all but a few bony fishes have another Opn5 homolog, Opn5m2, which forms a sister group with Opn5m (24). Opn5L1 exhibits considerably different molecular characteristics from the others, as it binds to all-*trans*-retinal (ATR) exclusively, becomes active, inactivates by visible light, and reverts spontaneously to the active state (25). In contrast, Opn5m, Opn5m2, and Opn5L2 bind to 11-*cis*-retinal (11CR) and form the inactive dark state which has maximum absorption in the ultraviolet (UV) wavelength region. Upon light absorption, 11CR is isomerized to ATR, leading to the formation of the active state (24, 26, 27, 28). Therefore, Opn5m is a common UV sensor highly conserved in vertebrates including humans.

In order to understand the molecular characteristics of Opn5m, coupled heterotrimeric G protein was analyzed in several previous studies (26, 27, 29, 30, 31, 32). Detergent-solubilized mouse and chicken Opn5m have been shown to activate Gi-type G proteins in the [^35^S]GTPγS filter–binding assay (26, 27). In fact, experiments in the mouse hypothalamus showed a light-dependent decrease of cAMP in Opn5-expressing cells, showing that Opn5 activates Gi in physiologically relevant conditions (29). In contrast, assays using cultured cells and *Xenopus* oocytes have shown that Opn5 induces an intracellular calcium response and depolarization of membrane potential, while [^35^S]GTPγS filter–binding assays could not detect the activation of Gq by chicken Opn5m (27, 30, 31). Even with this discrepancy, taken together with Opn5-dependent neuronal activation of quail hypothalamic cells (32), Opn5 might induce a calcium response in the cellular environment. The discrepancy is possibly because Opn5m causes a calcium response through the Gβγ-dependent pathway (33, 34), the PLC-IP_3_-Ca^2+^ system driven by Gα11, Gα14, or Gα15 rather than tested Gαq or other unknown system. In addition, the effect of the environment surrounding Opn5m and G protein in the respective assays and the variation in molecular properties of Opn5m orthologs from different species may also be a cause for the discrepancy.

To tackle this problem, we investigated Opn5m proteins from several separate species using an aequorin luminescence assay and a Gα-KO cell line. The Gα-KO cell line enabled us to investigate target Gα of Opn5m precisely in the uniform assay format and cell environment. The results show that the calcium response induced by light-activated Opn5m is dependent on Gq-type trimeric G proteins and that Opn5m preferentially activates Gα14, a member of the Gq-type Gα family, than the other Gq-type Gα proteins including canonical Gαq. To gain insight into the mechanism of this preferential activation of Gα14 by Opn5m, the experiments using point mutants/chimeras of mouse Gαq and Gα14 were performed, which showed that the efficiency of activation of the Gq-type G protein by Opn5m is affected by the amino acids in the region including α3-β5 and αG-α4 loops, αG and α4 helices, and the extreme C terminus. Taken together with the fact that another Gq-coupled opsin, jumping spider rhodopsin (JSR), did not show the preference for activation, Opn5m would have a mechanism to distinguish Gαq and Gα14. In addition to the calcium response, Opn5m would preferentially trigger downstream signaling specific to Gα14 than those specific to other Gq-type Gα proteins in Gα14-positive cells upon exposure to UV light. Co-expression of *opn5m/OPN5M* and *gna14a/GNA14* was found in the scleral cartilage of the medaka and chicken eyes by fluorescent *in situ* hybridization (FISH), while *opn5m/OPN5M-*positive cells in the neural retina were *gna14a/GNA14*-negative. This suggests the physiological relevance of preferential coupling of Gα14-Opn5m.

## Results

### Opn5m proteins trigger an intracellular calcium response

**Figure 1:**
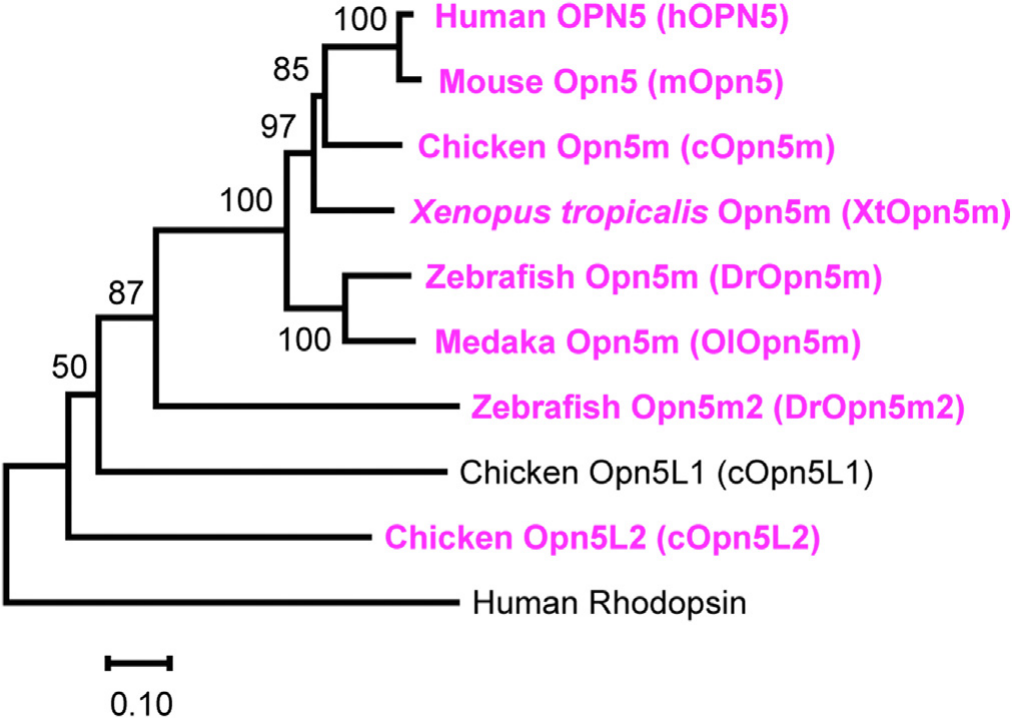
**Phylogenetic tree of Opn5 proteins investigated in this study.** The phylogenetic tree was inferred by the neighbor-joining method based on the aligned amino acid sequences. Human rhodopsin was included as an outgroup. Alignment of the amino acid sequences and construction of the tree were performed by MAFFT and MEGAX64, respectively (88, 92). Proteins analyzed in Figure 2 are highlighted in *magenta-*colored letters. The accession numbers of the amino acid sequences were as follows: human OPN5, NP_859528; mouse Opn5, NP_861418; chicken Opn5m, NP_001124215; *Xenopus tropicalis* Opn5m, XP_002936036; zebrafish Opn5m, NP_001186975; medaka Opn5m, XP_023808946; zebrafish Opn5m2, NP_001304680; chicken Opn5L1, NP_001296985; chicken Opn5L2, NP_001156364; and human rhodopsin, NP_000530. Opn5, opsin 5; Opn5m, mammalian type Opn5.

**Figure 2:**
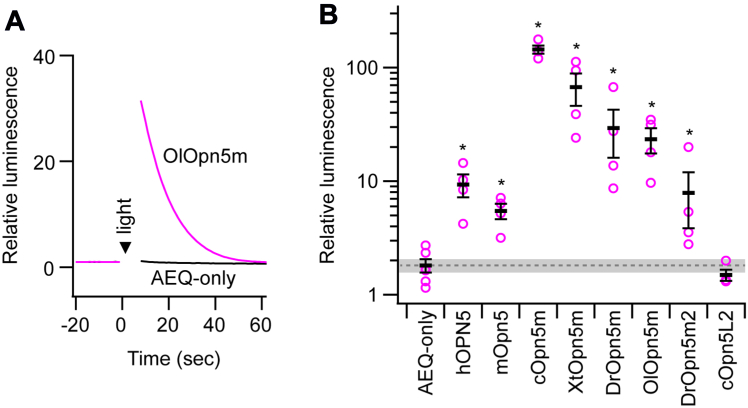
**Opn5m proteins trigger intracellular calcium response.** Intracellular calcium response investigated by aequorin luminescence assay in WT 293T cells. *A*, representative luminescence change of aequorin in OlOpn5m (*magenta curve*) and aequorin-only transfected (*black curve*) 293T cells. Luminescence intensity was normalized to values immediately before light irradiation (0 s). *B*, relative luminescence values calculated by dividing the luminescence intensity values just after UV light irradiation by those immediately before light irradiation. Error bars show SEM. *Magenta* plots show the individual data points. *Gray dotted line* and *shading* indicate the mean ± SEM of aequorin (AEQ)-only transfection control. Dunnett’s test was used for comparison to aequorin-only transfection (∗*p* < 0.05). Source raw luminescence traces were indicated in Fig. S13. Opn5m, mammalian type Opn5.

In this study, we analyzed Opn5m orthologs from separate taxonomic animal groups: mammals (human and mouse), bird (chicken), amphibian (*Xenopus tropicalis*), and teleosts (zebrafish and medaka). We also analyzed zebrafish Opn5m2 (DrOpn5m2) and chicken Opn5L2 (cOpn5L2), which are UV-sensitive bistable opsins phylogenetically close to Opn5m (Figs. 1 and S1) (24, 28). Opsins were cotransfected with a mitochondrial-targeted aequorin in the wildtype (WT) 293T cell line and were reconstituted with ATR supplied in a culture medium. Upon UV light irradiation, Opn5m proteins tested in this study induced a transient increase in the luminescence intensity of aequorin, which indicates that activation of Opn5m proteins leads to an increase in intracellular calcium concentration (Fig. 2, *A* and *B*). Likewise, DrOpn5m2 induced a light-dependent increase in the luminescence intensity (Fig. 2*B*). In contrast, cOpn5L2 showed an elevation of the luminescence level equivalent to that of the aequorin-only transfected control (Fig. 2*B*), though the UV irradiation was sufficient to decrease cellular cAMP level, probably by inhibition of adenylate cyclase through the heterotrimeric Gi (Fig. S2). We also examined these responses in the samples reconstituted with 11CR (Fig. S3), finding that Opn5m proteins and DrOpn5m2 triggered calcium responses, while cOpn5L2 did not. Responses in 11CR-reconstituted samples were generally more intense than those of ATR-reconstituted ones, which was prominent in human OPN5 (hOPN5), mouse Opn5, and DrOpn5m2. This would be related to the loss of direct ATR-binding ability of these proteins (24, 35). To confirm that the observed luminescent increase was truly derived from calcium-bound aequorin, we next used a cytosolic calcium chelator 1,2-bis(o-aminophenoxy)ethane-N,N,Nʹ,Nʹ-tetraacetic acid tetra(acetoxymethyl) ester (BAPTA-AM). Luminescent increase of aequorin by medaka Opn5m (OlOpn5m) was abolished in the presence of BAPTA-AM (Fig. S4*A*). Since opsin is a GPCR, the increase of calcium concentration upon light activation of Opn5m and Opn5m2 would be primarily mediated by the endogenous heterotrimeric G proteins in 293T cells.

### Knockout of GNAQ and GNA11 abolishes the calcium response by Opn5m

**Figure 3:**
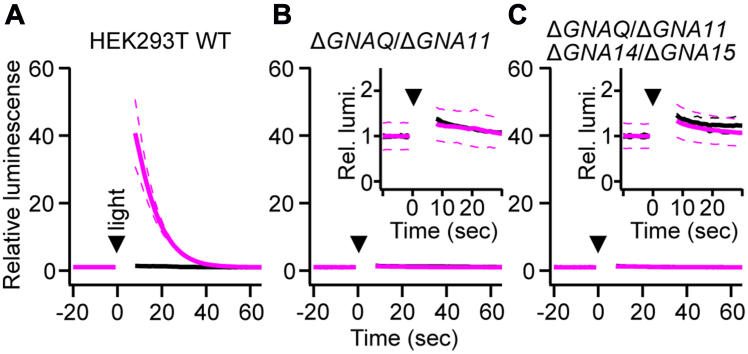
**Knockout of *GNAQ* and *GNA11* abolishes the calcium response by Opn5m.** Aequorin luminescence assay of OlOpn5m regenerated with ATR performed in 293T WT (*A*), *GNAQ/GNA11* double KO (*B*), and *GNAQ/GNA11/GNA14/GNA15* quadruple KO (*C*) cell lines. *Solid* and *broken curves* in *magenta* show the averaged and the individual experimental data, respectively (n = 2). Insets in the *middle* and *right panels* show the enlarged views. *Black traces* are negative control data with transfection of aequorin only. ATR, all-*trans*-retinal; Opn5m, mammalian type Opn5.

To determine the subtype of G proteins coupled with Opn5m which induce the calcium response, we performed genetic ablation of Gα proteins from 293T by CRISPR/Cas9 genome editing (Fig. S5). The previous studies have shown that WT 293 express *GNAQ* and *GNA11*, not *GNA14* and *GNA15*, and that genetic loss of *GNAQ* and *GNA11* is sufficient to suppress the Gq-PLC-IP3-Ca^2+^ system in 293 cells (36, 37). We first generated *GNAQ/GNA11* double knockout (KO) 293T cells (Δ*GNAQ*/Δ*GNA11*). The aequorin assay performed on OlOpn5m showed that the calcium response seen in WT cells was completely absent in Δ*GNAQ*/Δ*GNA11* cells (Fig. 3, *A* and *B*). In this study, to avoid the possibility of unexpected upregulation of *GNA14* or *GNA15* expression by transfection of exogenous genes and/or by addition of reagents such as retinal, quadruple KO cells, in which *GNA14* and *GNA15* were also knocked out, were generated and investigated (Fig. 3*C*). As shown in the double KO Δ*GNAQ*/Δ*GNA11* cells, the quadruple KO cells also showed no calcium response even with transfection of medaka Opn5m and supply of ATR. This quadruple KO (Δ*GNAQ*/Δ*GNA11*/Δ*GNA14*/Δ*GNA15*) cell line is used hereafter and referred to as 293TΔGQ below.

### Gq-type Gα subunit can rescue the calcium response by Opn5m in 293TΔGQ cells

**Figure 4:**
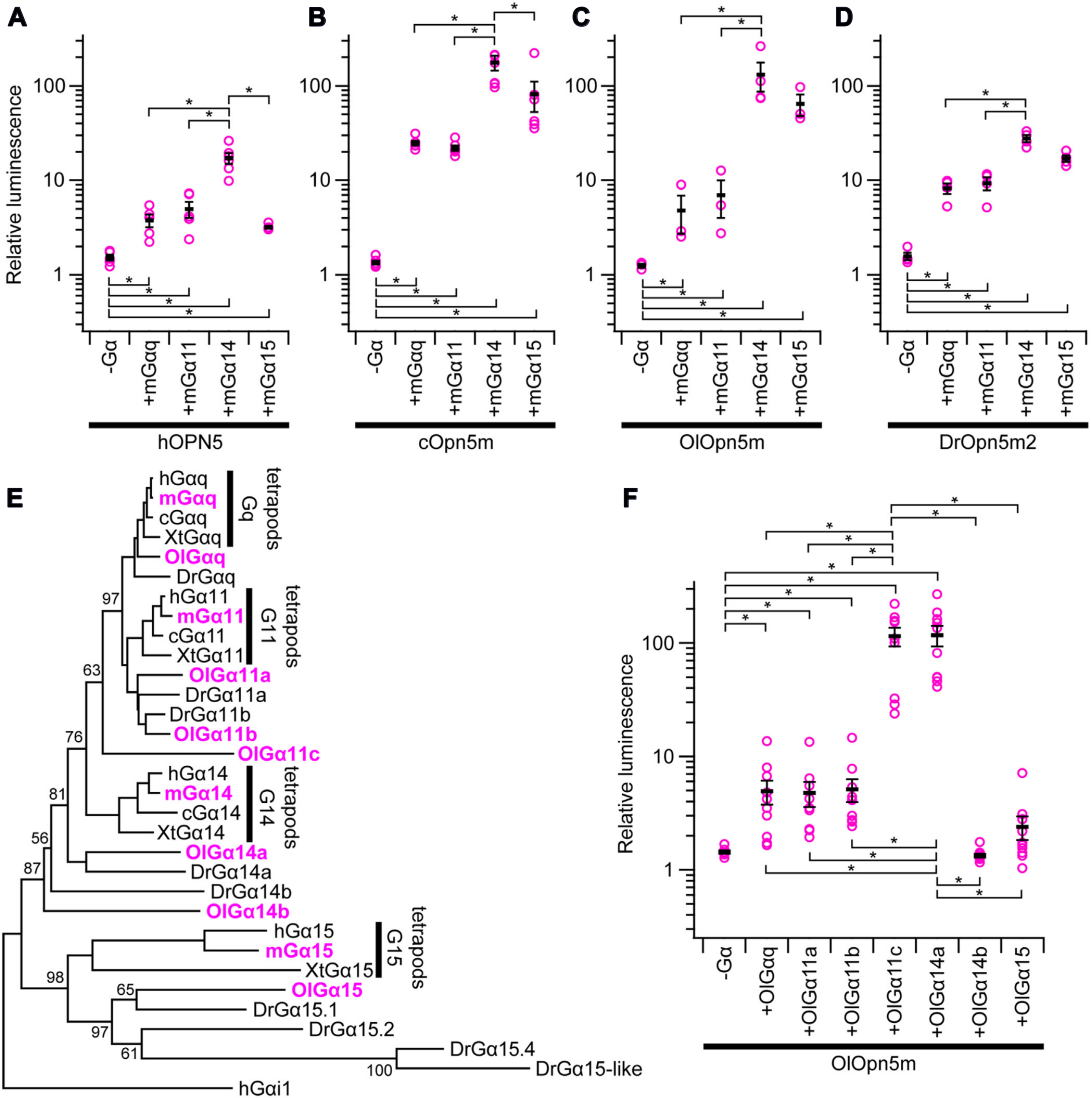
**G14 is preferentially activated by Opn5m.** Aequorin luminescence assay of Opn5 proteins performed in the 293TΔGQ cell line. *A*–*D*, light-dependent increase of luminescence was analyzed in the cell transfected with hOPN5 (*A*), cOpn5m (*B*), OlOpn5m (*C*), or DrOpn5m2 (*D*) in combination with or without mouse Gαq, Gα11, Gα14, and Gα15. Sequence identity to mGαq is 89.1%, 81.4%, and 57.0% for mGα11, mGα14, and mGα15, respectively. Relative luminescence values calculated by dividing the luminescence intensity values just after UV light irradiation by those immediately before light irradiation. Individual data points are shown in *magenta*. Error bars show SEM. The Tukey–Kramer test was used for multiple comparison (∗*p* < 0.05). *E*, the phylogenetic tree inferred by the neighbor-joining method based on the amino acid sequences of Gq-type G protein alpha subunits. Human Gαi1 (hGαi1) was included as an outgroup. The alignment of the amino acid sequences and construction of the tree were performed by MAFFT and MEGAX64, respectively (88, 92). Gα proteins from mouse and medaka were highlighted in *magenta*. The accession numbers of the amino acid sequences included in this tree were as follows: human Gαq (hGαq), NP_002063.2; human Gα11 (hGα11), NP_002058; human Gα14 (hGα14), NP_004288; human Gα15 (hGα15), NP_002059; mouse Gαq (mGαq), NP_032165; mouse Gα11 (mGα11), NP_034431; mouse Gα14 (mGα14), NP_032163; mouse Gα15 (mGα15), NP_034434; chicken Gαq (cGαq), NP_001026598; chicken Gα11 (cGα11), NP_989565; chicken Gα14 (cGα14), XP_046791757; *Xenopus tropicalis* Gαq (XtGαq), NP_001037982; *X. tropicalis* Gα11 (XtGα11), NP_989150; *X. tropicalis* Gα14 (XtGα14), NP_001008054; *X. tropicalis* Gα15 (XtGα15), XP_002934852; zebrafish Gαq (DrGαq), NP_001138271; zebrafish Gα11a (DrGα11a), NP_001038501; zebrafish Gα11b (DrGα11b), NP_001007774; zebrafish Gα14a (DrGα14a), XP_683989; zebrafish Gα14b (DrGα14b), NP_001003753; zebrafish Gα15.1 (DrGα15.1), NP_001003626; zebrafish Gα15.2 (DrGα15.2), XP_002667410; zebrafish Gα15.4 (DrGα15.4), NP_001038454; zebrafish Gα15-like (DrGα15-like, si:ch211-207c7.2), XP_003201106; medaka Gαq (OlGαq), XP_023814176; medaka Gα11a (OlGα11a), XP_004068399; medaka Gα11b (OlGα11b), XP_020569601; medaka Gα11c (OlGα11c), XP_023816641; medaka Gα14a (OlGα14a), XP_011477393; medaka Gα14b (OlGα14b), XP_004074607; medaka Gα15 (OlGα15) XP_023810335; and hGαi1, NP_002060. *F*, light-dependent increase of luminescence was analyzed in the cells introduced of OlOpn5m in combination with or without respective medaka Gα proteins. Relative luminescence values were calculated by dividing the luminescence intensity values just after UV light irradiation by those immediately before light irradiation. *Magenta plot* shows the individual data points. The *black bars* and error bars show mean ± SEM. The Tukey–Kramer test was used for multiple comparison (∗*p* < 0.05). Source raw luminescence traces were indicated in Fig. S13. hOPN5, human OPN5; Opn5, opsin 5; Opn5m, mammalian type opsin 5.

Next, an aequorin calcium assay with cotransfection of Opn5 and a mouse Gq-type Gα subunit was performed on 293TΔGQ cells. Here, hOPN5, cOpn5m and OlOpn5m, and DrOpn5m2 were investigated as representatives of mammalian Opn5, nonmammalian Opn5m, and Opn5m2, respectively. First, in the absence of cotransfection of Gα in 293TΔGQ cells, none of the opsins tested here generated a light-dependent calcium response seen in WT 293T cells even with reconstitution of ATR or 11CR (Figs. 4, *A*–*D*, S6). Also, addition of BAPTA-AM did not cause any change in relative luminescence without transfection of Gα (Fig. S4*B*). This shows that photoactivation of Opn5m does not affect intracellular calcium level in the absence of Gq-type Gα. With cotransfection of Gα, a light-dependent calcium response was significantly observed in any combination of these four opsins, four subtypes of mouse Gq-type α subunits, although there were differences in the intensity (Figs. 4, *A*–*D* and S6). In all hOPN5, cOpn5m, OlOpn5m, and DrOpn5m2 cases, the response was maximal when mGα14 was cotransfected. The difference between mGαq and mGα14 was the largest for ATR-reconstituted OlOpn5m, for example, with a 27-fold difference in relative luminescence (Fig. 4*C*).

We next performed an assay using Gq-type α subunit from medaka with OlOpn5m (Fig. 4, *E* and *F*). Since some teleost Gq-type Gα proteins are not included in well-separated clusters of Gαq, Gα11, Gα14, and Gα15, which are tetrapod Gq-type α subunits in the phylogenetic tree, it was intriguing to investigate whether there were Gα proteins preferentially activated by Opn5m in teleosts (Fig. 4*E*). This exploration could also provide insights into the specific amino acid sequences that Opn5m recognizes to distinguish Gα14 from the other subunits. We performed the aequorin calcium assay in 293TΔGQ cells. OlGαq, OlGα11a, and OlGα11b showed moderate response intensity, while OlGα11c and OlGα14a showed a ∼24-fold more intense response, which is like that seen in the comparison between mGαq and mGα14 (Fig. 4*F*). The luminescence increases in OlGα14b and OlGα15 were either very small or indistinguishable from the negative control (Fig. 4*F*). These observed differences in amplitude of calcium response by Opn5m can be attributed to several factors such as expression level of functional recombinant Gα, intrinsic ability of G proteins to trigger calcium response, and efficacy of receptor-G protein coupling. To test these possibilities, the relative expression levels of recombinant Gα-forming native protein complex including heterotrimer were estimated by Western blot after native PAGE based on the previous reports (Figs. S7 and S14, Table S3) (38, 39). These results showed that relative expression levels of functional recombinant Gα tested in this study were comparable to each other except for OlGα14b, showing a lower expression level (Fig. S7, *Q* and *R*). Additionally, we performed an aequorin assay with JSR, another Gq-coupled opsin, to confirm receptor-specific variation of Gα activation and to validate the functionality of OlGα14b and OlGα15 (Fig. S8) (40). Consequently, JSR induced a comparable light-dependent luminescent increase in all mouse and medaka Gα tested in this study including OlGα14b and OlGα15. Thus, the difference in calcium response observed in Figure 4 would be attributed at least in part to the preference of Opn5m-G protein coupling.

### Analysis of chimeras and point mutants of mGαq and mGα14

**Figure 5:**
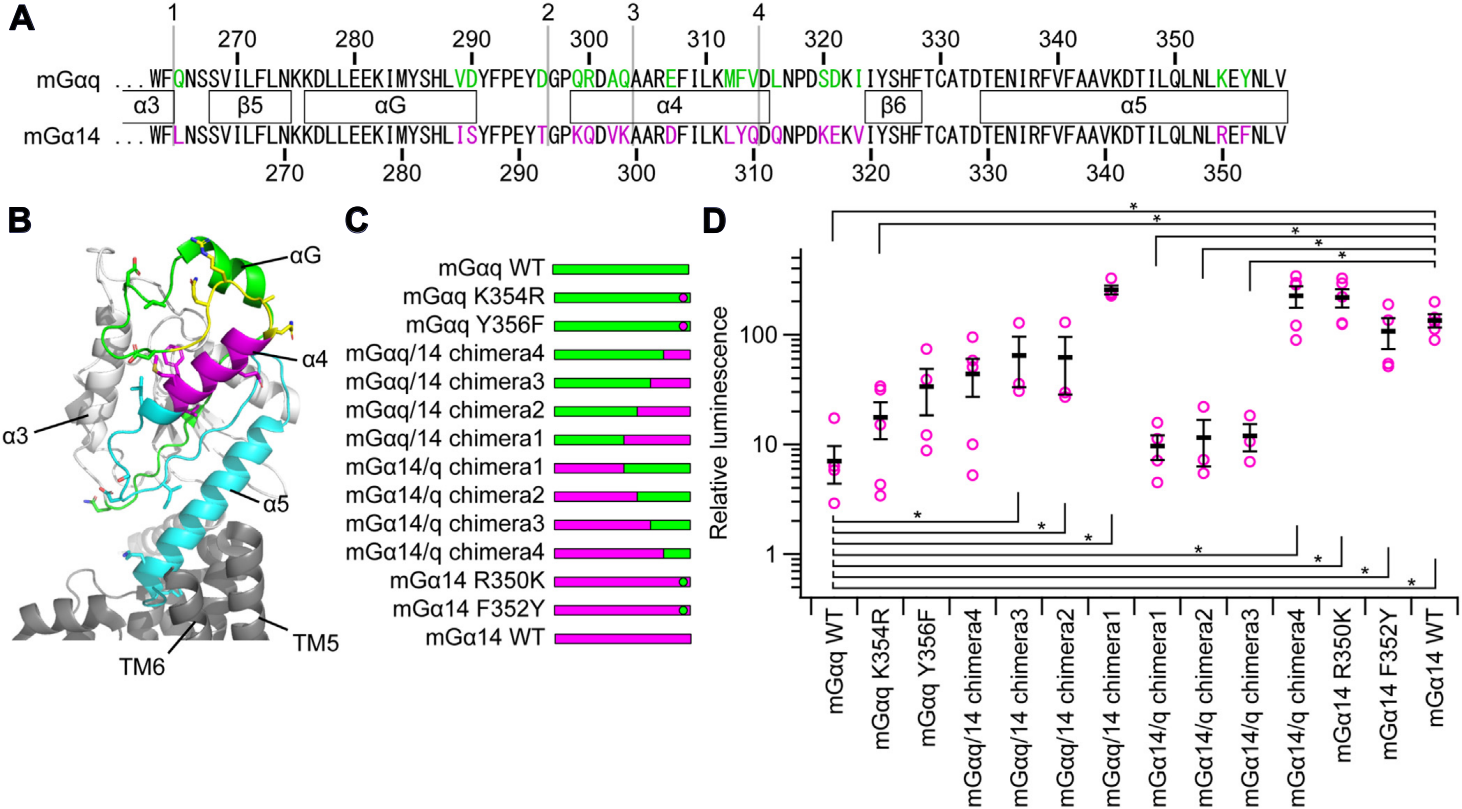
**Activation of chimeras and point mutants of mGαq and mGα14 by Opn5m.***A*, amino acid sequence alignment of mGαq and mGα14. The residue number of mGαq is shown above the sequences, while that of mGα14 is shown below. The numbered *gray lines* indicate the fusion positions of mGαq and mGα14 chimeras. Amino acids that differ between mGαq and mGα14 are highlighted in *green and magenta*. Secondary structural features of mGαq are denoted between the two sequences. *B*, the positions of chimeric structures depicted utilizing the cholecystokinin B receptor (CCKBR)–Gq complex structure (pdb:7f8w). CCKBR is drawn in *gray*. In Gαq, N-terminal region from position 1, the regions between position 1 and 2, 2 and 3, 3 and 4, and C-terminal region from position 4 are drawn in *white*, *green*, *yellow*, *magenta*, and *cyan*, respectively. *C*, the chimera and mutant constructions depicted schematically with *green* regions derived from mGαq and *magenta* from mGα14. *D*, light-dependent increase of luminescence was analyzed in the cells introduced of OlOpn5m in combination with or without respective chimeric Gα proteins. Relative luminescence values were calculated by dividing the luminescence intensity values just after UV light irradiation by those immediately before light irradiation. *Magenta plot* shows the individual data points. The *black bars* and error bars show mean ± SEM. The Tukey–Kramer test was used for multiple comparison (∗*p* < 0.05). Significant differences from mGαq WT and mGα14 WT are shown. Source raw luminescence traces were indicated in Fig. S13.

To gain insight into the mechanism of preferential activation of Gα14 among Gq-type Gα proteins by Opn5m, we performed experiments using point mutants and chimeric proteins of mGαq and mGα14 with ATR-reconstituted OlOpn5m in 293TΔGQ cells. Since the main interface of Gα to the receptor is the C-terminal α5 helix (41, 42), the extreme C-terminal amino acids that differ in mGαq and mGα14 were replaced with the corresponding residues in the other. Additionally, chimeric proteins mGαq/14 and mGα14/q were created by fusing the N-terminal region of mGαq to the C-terminal region of mGα14 (mGαq/14 chimeras) or the N-terminal region of mGα14 to the C-terminal region of mGαq (mGα14/q chimeras) at four different positions (Figs. 5, *A*–*C* and S9*A*). The expression levels of these mutants and chimeras were confirmed to be comparable to those of mGαq and mGα14 (Fig. S7, *S* and *T*).

Compared to mGαq WT, the K354R and Y356F point mutants showed a slight increase in the relative luminescence intensity in an aequorin calcium assay (Fig. 5*D*). In mGαq/14 chimeras, chimera 4 showed relative luminescence equivalent to that of mGαq Y356F, suggesting that the amino acid substitutions of L316Q, S320K, D321E, and I323V in mGαq/14 chimera 4 do not affect the interaction with OlOpn5m. Those of chimeras 2 and 3 were equivalent to each other and significantly more than that of mGαq WT. Furthermore, mGαq/14 chimera 1 showed a greater relative luminescence than chimeras 2 and 3. These results imply that the substitutions of Q265 in the α3-β5 loop; V289 and D290 in the αG helix; D296 in the αG-α4 loop; and E307, M312, F313, and V314 in the α4 helix into the corresponding amino acids in mGα14 cooperatively contribute to the preferential activation by OlOpn5m (Fig. 5, *A* and *B*). Next, in mGα14 point mutants, the difference between the relative luminescence of mGα14 WT and those of mGα14 R350K and F352Y were not significant, while they were slightly increased and reduced compared to that of WT, respectively (Fig. 5*D*). In mGα14/q chimeras, chimera 4 showed a response comparable to WT mGα14. However, the relative luminescence intensities of chimeras 1, 2, and 3 were reduced to about 0.08-fold of WT (Fig. 5*D*). This suggests that D303, L308, Y309, or Q310 in the α4 helix is necessary for the preferential activation of mGα14 by OlOpn5m (Fig. 5, *A* and *B*). Among these residues, D303 in the α4 helix is found also in both OlGα11c and OlGα14a, those preferentially activated by OlOpn5m in medaka Gq-type α subunits though the latter three residues in OlGα11c are identical to those in mGαq (Fig. S9*A*).

### Identification of the cells expressing both Opn5m and Gna14

**Figure 6:**
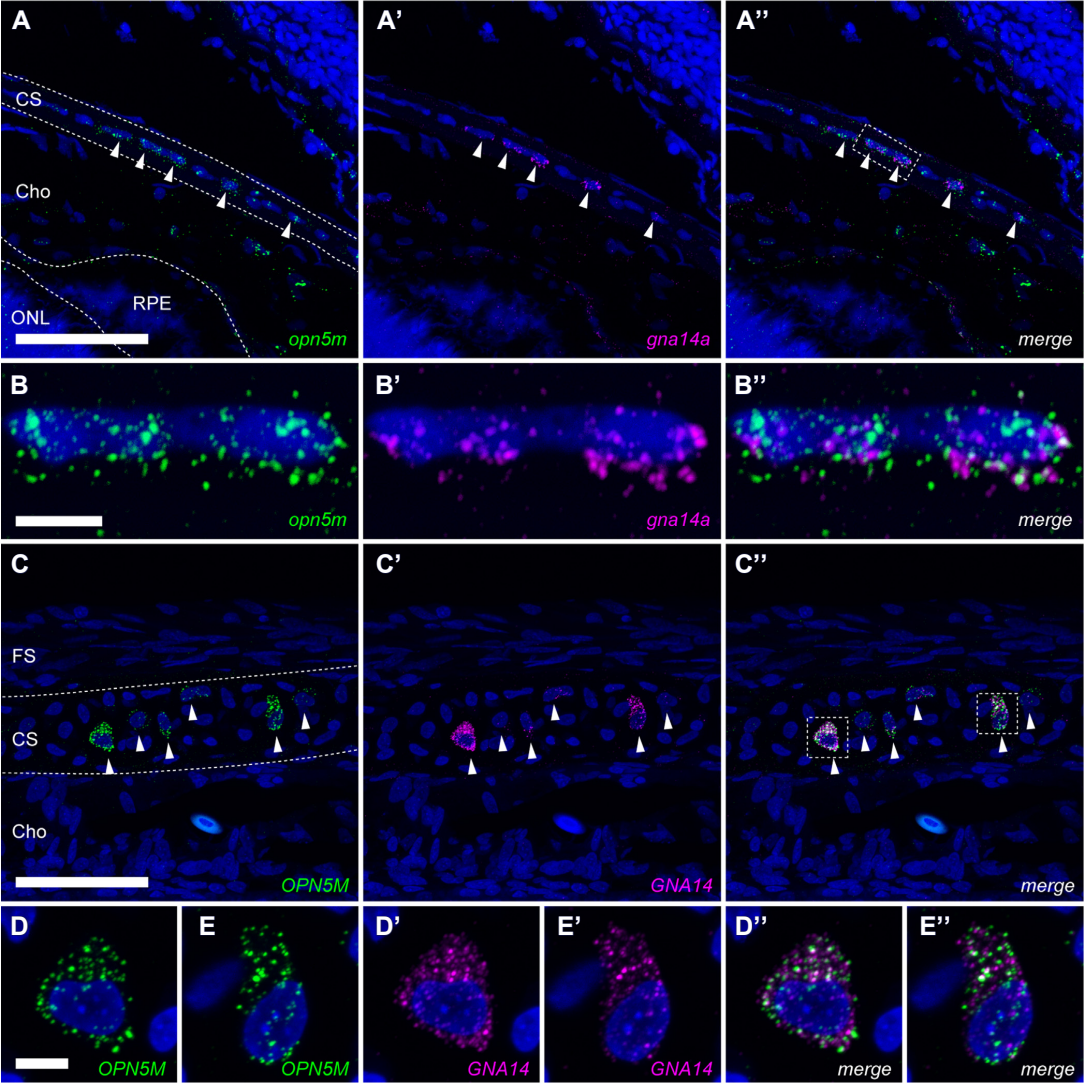
**Distribution of mRNA of *opn5m/OPN5M*and *gna14a/GNA14*in the medaka and chicken eyes.** Fluorescent *in situ* hybridization using signal amplification by exchange reaction (SABER). Medaka eye (*A* and *B″*) and chicken eye (*C*–*E″*) were assessed. Fluorescent signals of medaka *opn5m* (*A* and *B*), *gna14a* (*A′* and *B′*), chicken *OPN5M* (*C*–*E*), and *GNA14* (*C′*, *D′* and *E′*) are shown. *White arrowheads* indicate co-expression of the two genes in the scleral cartilage of medaka and chicken eyes. *Broken curves* in *panels A* and *C* show the boundaries of different histological areas. *Broken boxes* in *panels A″* and *C″* indicate the regions enlarged in *panels B*-*B″*, *D*-*D″*, and *E*-*E″*. *Panels A″*, *B″*, *C″*, *D″*, and *E″* show the merged images. Cell nuclei were stained by Hoechst 33342 (*blue*). Images are maximum intensity projections of confocal z-stacks. The scale bars in *A* and *C* represents 50 μm; and the scale bars in *B* and *D* represents 5 μm. Cho, choroid; CS, cartilaginous sclera; FS, fibrous sclera; ONL, outer nuclear layer; Opn5m, mammalian type Opn5; RPE, retinal pigment epithelium.

Finally, as a clue to understand the physiological significance, we sought to find the cells having Opn5m along with Gα14 in animal tissues. Because medaka eye tissue showed relatively high level of expression of *gna14a* compared to other neural tissues (Fig. S10*E*), distribution of mRNA was explored with signal amplification by exchanging reaction (SABER)-FISH in medaka and chicken eye tissues (Figs. 6 and S11) (43). mRNA signals for *opn5m/OPN5M* were detected in the inner nuclear layer and ganglion cell layer of the medaka and chicken retinas, but not for *gna14a/GNA14* in two color SABER-FISH on the retina (Fig. S11, *E*-*E*’’, *F*-*F*’’). In contrast, a subset of chondrocytes in medaka and chicken scleral cartilage expressed both genes (Fig. 6). The fluorescent signals of mRNAs were observed in the peripheral region of medaka eyes and in the posterior pole region of chicken eyes (Fig. S11, *A*–*D*). These results suggest that those scleral chondrocytes are UV-sensitive and induce an intracellular response through G14-subtype Gα protein. In medaka eyes, *gnaq*, *gna11a*, and *gna11b* are abundantly expressed in the inner nuclear layer and ganglion cell layer (Fig. S10). These subtypes would work with Opn5m in the inner nuclear layer and ganglion cell layer to generate modest response. The difference in responses to UV light would be related to the functional difference in UV sensing.

## Discussion

Using an intracellular aequorin assay, this study shows that the calcium response triggered by light-activated Opn5m is abolished by genetic ablation of *GNAQ/GNA11* and restored by reconstitution with Gq-type Gα proteins. Taken together with the fact that depletion of intracellular calcium stores by thapsigargin and inhibition of Gαq/11 by YM-254890 abolish the calcium response (30, 44), it seems plausible that Opn5m generally activates G proteins in the Gq family and leads to the calcium response. Live-cell assays and biochemical assays using extracted and purified proteins differ in numerous factors that affect the activity of GPCRs. The membrane environment, including surfactant and lipids, largely affects receptor activity (45, 46). Therefore, it is possible that the ability to activate Gαq was lowered in detergent-purified cOpn5m, making it undetectable in GTPγS filter–binding assays (27).

In mammals, *GNAQ* and *GNA11* are expressed ubiquitously, while *GNA14* is reported to be expressed in spleen, lung, kidney, testes, etc., and *GNA15* is especially expressed in hematopoietic cells (47, 48). These Gαq, Gα11, Gα14, and Gα15 all activate PLCβ (49, 50), which induces DAG-dependent protein kinase C activation and IP3-induced intracellular calcium responses. Though the examples of functional difference in the subclasses of Gq-type Gα proteins are presented in the introduction, the mechanism by which they differently function remains to be elucidated. In our aequorin assay, Opn5 activated all the subtypes of mouse Gq-type α subunits, with Gα14 showing the highest level of activation. Several GPCRs, such as the thromboxane A2 receptor alpha isoform, have also been reported to activate Gαq, Gα11, Gα14, and Gα15 with different efficiencies, implying that distinct preferences in Gq-type Gα proteins to diverse downstream functions are linked to the receptor functions (51, 52). Conversely, the receptors that strongly activate a particular subtype by light, like Opn5m, may be an effective tool to investigate different functional properties of trimeric G protein subtypes.

As for the physiological functions of Opn5, it has been shown to be involved in light entrainment of local circadian rhythms in the retina, cornea, and skin (53, 54); regulation of vitreous blood vessels during development (55); and suppression of heat production in brown adipocytes through photoreception in the hypothalamus in mice (29). In quail, the paraventricular organ is involved in light reception and day-length perception (31, 32). We found co-expression of *opn5m/OPN5M* and *gna14a/GNA14* in the scleral cartilage of medaka and chicken eyes, which would be related to a currently unidentified photoreceptive function. The expression of Opn5m in the cartilaginous sclera of the chicken eye is consistent with our previous observation by RT-PCR (56). The co-expression of *opn5m/OPN5M* and *gna14a/GNA14* in scleral chondrocytes implies that, like cytokines (57), hormones (58), and mechanical stress (59, 60), UV light might affect chondrocyte survival, proliferation, differentiation, or production of cartilaginous extracellular matrix in the sclera. In humans, thinning of the sclera and choroid is seen in high axial myopia (61). Several studies suggested that violet light is suppressive to myopic development of eye axis length (62, 63). Additionally, it has been shown that myopia suppression by violet light is mediated by Opn5 in mice (64). These observations suggest the presence of a conserved relationship between control of ocular axis length and short-wavelength light in vertebrate species, although there would be different mechanisms in mammals and other vertebrates because the expression of Opn5 in mouse eyes is restricted to ganglion cells and mammals do not have cartilage in the sclera. In our experiment, *gna14a/GNA14* could not be detected in the neural retinal cells of the medaka or chicken retina, such as amacrine and ganglion cells, where *opn5m/OPN5M* could be found. This shows that Opn5m in those cells functions through activation of ubiquitous Gαq/11 and/or Gαi. The difference between those *opn5m*/*gna14a* double positive and *opn5m*-positive/*gna14*-negative cells may be helpful for a future study to understand the physiological significance of biased signaling in the subtypes of Gαq by Opn5m.

Previously reported Gq-controlling optogenetics tools are melanopsin (65, 66, 67) and Opto-XR–type chimeric photosensitive proteins (68, 69). Based on our observation, Opn5m can be used as an optogenetic tool to control the intracellular Gq pathway by UV light. In fact, Opn5m has recently started to be used as the optogenetic tool to control Gq signaling (44, 70). In the current context, where no practical, calcium-selective channel rhodopsin has yet been found or produced, Opn5 may be an option as a tool to optogenetically manipulate the intracellular calcium concentration. Because Opn5m is UV-sensitive, multicolor simultaneous control of multiple heterotrimeric G proteins would be enabled in combination with visible light–sensitive Gi-coupled and Gs-coupled opsins such as vertebrate visual opsins and jellyfish opsins (71, 72).

Through the experiments involving point mutants and chimeras of mGαq and mGα14, we have shown that up to ten amino acids in the region including α3-β5 and αG-α4 loops, αG and α4 helices, and the extreme C terminus modulate the coupling efficiency with Opn5m to generate a 27-fold difference in the intensity of the calcium response. The C terminus of Gα has been identified as critical for activation by the receptor and as an important determinant of selectivity (73, 74, 75). The C-terminal α5 helix is wrapped around the transmembrane domain of the GPCR during coupling (76, 77). Additionally, recent studies have shown that different GPCRs can recognize different features of the same G protein for selective coupling (78). In addition to low expression level of OlGα14b, OlGα14b and OlGα15 have the amino acid sequences, “GLE” and “GVM,” deviated from “NLV” conserved in the others at the extreme C terminus that are considered most important for interaction with GPCR (Fig. S9*A*) (73, 74). This would also lower the preference from Opn5m in the case of OlGα14b and OlGα15. In our experiments using medaka Gq-type Gα, OlGα14b and OlαG15 were activated efficiently by JSR, but not by OlOpn5m. These promiscuous or biased activations of Gq-type Gα proteins by JSR or OlOpn5m show that the mechanism for determining Gα protein preference is different in these two opsins, although they are both opsins that activate Gq-type Gα proteins. Using these findings, further studies may allow us to create a highly bio-orthogonal cellular function regulating tool, such as a photoactivatable GPCR that stimulates artificial G protein without affecting endogenous G proteins at all.

In summary, Opn5m of several vertebrate species could activate Gαq, Gα11, Gα14, and Gα15 in response to UV light and trigger an increase of intracellular calcium concentration. Among these Gq-type Gα proteins, calcium response by Opn5m was the most intense in Gα14. Difference in several specific amino acids in the region including the α3-β5 and αG-α4 loops, the αG and α4 helices, and the extreme C terminus causes the difference in the activation efficiency of Gα14 and Gαq by Opn5m. Additionally, it was found that Opn5m2 activated Gαq-type G proteins like Opn5m, while Opn5L2 did not, although they are phylogenetically close and all sensitive to UV light, showing that Opn5L2 is coupled to a different profile of downstream pathways. Finally, we found cells expressing both *opn5m/OPN5M* and *gna14a/GNA14* in medaka and chicken eyes, which shows that preferential coupling of Opn5m and Gα14 protein has some functional relevance.

## Experimental procedures

### Plasmid construction

Opsin complementary deoxyribonucleic acids (cDNAs) inserted in the pCAGGS vector were constructed in previous studies (24, 28, 35) except for JSR (40). Human codon–optimized JSR was synthesized by GeneArt Strings DNA Fragments (Thermo Fisher Scientific), PCR-amplified, and inserted into pCAGGS. All opsins were tagged with rho1D4 epitope (ETSQVAPA) at the C terminus (24, 28, 35). cDNAs for mouse and medaka Gq-type G protein alpha subunits were isolated by PCR using first-strand cDNA from adult mouse and medaka brains and inserted into pCAGGS. After repairing the reported mutation in the puromycin resistance gene (Puro) in PX459 plasmid (Addgene #48139), *Streptococcus pyogenes* Cas9 (SpCas9) linked with Puro *via* 2A peptide (SpCas9-2A-Puro) was amplified from PX459 by PCR and ligated to the pCAGGS vector. To construct a separate single guide RNA (sgRNA) expression vector, the region containing U6 promoter, BbsI restriction sites, and sgRNA scaffold sequence was amplified from PX459 by PCR and inserted into the pGEM-5Zf(+) vector. The target DNA for genome editing was prepared by annealing two DNA oligos and was ligated into a BbsI-digested sgRNA expression vector.

Mitochondrial-targeted aequorin N26D was amplified from pcDNA3.1+/mit-2mutAEQ (Addgene plasmid #45539) by PCR with repairing two mutations, L28N and A119D, to the WT amino acids and was ligated to the pCAGGS vector. Ligation of PCR products into vectors was performed by the SLiCE method (79, 80). pcDNA3.1+/mit-2mutAEQ and pSpCas9(BB)-2A-Puro (PX459) were gifts from Javier Alvarez-Martin and Feng Zhang through Addgene (http://www.addgene.org), respectively (81, 82).

### Generation of a Gα-KO cell line

Target sites for genome editing were chosen using the web tool CRISPRdirect (https://crispr.dbcls.jp/) to minimize the possibility of off-target cleavage (83). The information about the chosen target sequences is summarized in Fig. S5. 293T cells were seeded into 24-well plates in Dulbecco's modified Eagle's medium/F-12 (FUJIFILM Wako) containing 10% fetal bovine serum (FBS). After a day, cells were transfected with the SpCas9-2A-Puro expression plasmid and sgRNA expression plasmid using HilyMax (DOJINDO) according to the manufacturer’s instructions. Six to eight hours after transfection, the medium was replaced with a fresh medium. Twenty four hours after transfection, puromycin was supplied at 5 μg/ml. Twenty four hours after the addition of puromycin, live cells were trypsinized, counted, and seeded again on a 10-cm culture dish at 100 to 300 cells per dish. After 15 days, clonal colonies on the culture dish were scratched and transferred to 24-well plates. Genotyping was performed by PCR for each clone to identify the cells carrying biallelic Gα gene KO. The *GNAQ*/*GNA11* double–KO cell was generated by sequentially repeating the above process twice. Thereafter, the *GNAQ*/*GNA11*/*GNA14*/*GNA15* quadruple KO cell was produced by one step of the same procedure, involving sgRNA expression plasmids targeting both *GNA14* and *GNA15.*

### Aequorin luminescence–based calcium assay

A luminescent calcium assay using aequorin as an indicator was performed according to the previous reports (84, 85). 293T cells were seeded into white-walled 96-well plates (MS-8096W, Sumitomo Bakelite) at 20,000 cells per well in Dulbecco's modified Eagle's medium/F-12 (FUJIFILM Wako) containing 10% FBS. After a day, cells were transfected with the expression plasmids of the opsin and the mitochondrial targeted aequorin N26D using polyethylenimine (PEI MAX, Polyscience). The ratio of polyethylenimine:plasmid was 4:1 in weight. The ratio of plasmids of opsin:aequorin (Figs. 2 and 3) and that of opsin:Gα:aequorin (Figs. 4 and 5) were 1:1 and 1:1:2 in weight, respectively. Six to eight hours after transfection, the medium was replaced with a fresh medium containing 2 μM 11CR or 5 μM ATR. The next day, the medium was replaced with an L-15 medium without phenol red (Invitrogen) containing 10% FBS and 10 μM coelenterazine h (FUJIFILM Wako) under dim red light. After 2 h of incubation in the dark, luminescence was measured using a microplate luminometer (Veritas, Turner Biosystems). The cells were stimulated with a handheld UV flashlight (0.11 ± 0.0036 mW mm^−2^; peak irradiance at 373 nm, Fig. S12) for 5 s.

### Statistical analysis

Statistical analysis was performed for log-transformed values of relative luminescent intensities with Igor Pro 9 software (https://www.wavemetrics.com/products/igorpro) ver 9.0.2.4 (WaveMetrics).

### Animal tissue preparation

The use of animals in these experiments was in accordance with guidelines established by the Ministry of Education, Culture, Sports, Science and Technology of Japan. The animal experimental design was approved by the Animal Care and Use Committee, Okayama University. The medaka fish (*Oryzias latipes*) strain d-rR was maintained and bred at Okayama University. They were kept under a light/dark cycle of 14/10 h at 26 ± 1 °C. Mature medaka (>25 mm in body length) in light period, 4 to 12 h after light-on, were euthanized in 0.04% tricaine methanesulfonate and immediately decapitated. One-day-old male broiler chicks were purchased from Fukuda poultry breeding farm. They were euthanized by intraperitoneal injection of 150 mg/kg pentobarbital sodium. Regarding the chicks, cardiac perfusion of 4% paraformaldehyde in phosphate-buffered saline (PBS) was performed before dissection of the eyes. After the eyes were dissected from the medaka and chicks, they were fixed overnight in 4% paraformaldehyde in PBS at 4 °C. The fixed eyes were subsequently immersed in 20% sucrose in PBS overnight for cryoprotection and were frozen in optimum cutting temperature compound (Sakura Finetek) in a deep freezer at −80 °C. Frozen tissues were sliced into 15 μm sections with a cryo-microtome (Polar, Sakura Finetek) and were attached to glass slides (CREST-coated glass slide, Matsunami Glass). These glass slides were stored at −20 °C until use.

### Fluorescent *in situ* hybridization

Fluorescence detection of mRNAs of medaka *opn5m*, *gna14a*, chicken *OPN5M*, and *GNA14* in the eye tissue was performed with SABER according to the original paper (43). ssDNA probes and branches for signal amplification were extended by primer exchange reaction, employing BST DNA polymerase large fragment, DNA hairpin oligos, dATP, dTTP, and dCTP (86). Synthesized probes and branches were confirmed to be 400 to 700 nts in length with agarose gel electrophoresis and purified using DNA Clean & Concentrator-25 (Zymo Research).

The chicken and medaka eyes sectioned onto the glass slides were immersed in PBS containing 0.1% Tween 20 (PBST) for 15 min at room temperature (RT) and in Whyb buffer (0.3 M NaCl, 30 mM sodium citrate, 40% deionized formamide, pH 7.0) at 43 °C for 10 min. After that, probes diluted with Hyb1 buffer (Whyb buffer containing 10% dextran sulfate) at final concentration of 1 μg/ml each were applied onto the tissue sections, which were incubated for about 16 h at 43 °C. After hybridization, they were washed in Whyb buffer at 43 °C for 30 min twice, in 2X saline-sodium citrate with Tween 20 (SSCT) buffer (0.3 M NaCl, 30 mM sodium citrate, 0.1% Tween 20, pH 7.0) at 43 °C for 5 min twice, and rinsed with PBST twice at RT. After the immersion in Whyb at 37 °C for 10 min, the branch DNAs diluted with Hyb1 buffer at a final concentration of 1 μg/ml each were applied onto the tissue sections, which were incubated for about 16 h at 37 °C. After two washes in Whyb at 37 °C for 30 min, two washes in 2X SSCT buffer at 37 °C for 5 min, two rinses with PBST at RT, and a wash in Whyb at 37 °C for 10 min, the second-round amplification for 16 h at 37 °C with addition of the second branch DNA at 1 μg/ml each were carried out. After the identical series of washes with Whyb, 2X SSCT, and PBST, the tissue sections were incubated with oligo DNAs labeled by fluorophore diluted in PBST at 0.1 μM at 37 °C for 1 h. The tissue sections were rinsed twice with PBST and were counterstained with 1 μg/ml Hoechst 33342 in PBST. Finally, coverslips were mounted on glass slides with homemade polyvinyl alcohol/glycerol mounting medium.

Confocal fluorescence images were collected with a laser scanning confocal microscope system, Zeiss LSM 780 (Carl Zeiss Microscopy GmbH) with 405, 488, and 561 nm laser lines at the Central Research Laboratory, Okayama University Medical School. Confocal z-stack images were acquired at 0.540-μm intervals for a total depth of 14.04 (Fig. 6, *A*-*A’’*, *C*-*C’’*), 11.88 (Fig. 6, *B*-*B’’*), 7.56 (Fig. 6, *D*-*D’’*), or 8.64 (Fig. 6, *E*-*E’*’) μm. Manipulation of confocal fluorescence images was performed by ZEN 2012 SP1 black edition (https://www.zeiss.com/microscopy/en/products/software/zeiss-zen.html) (64-bit, version 8.1). DNA sequences of probes, branches, hairpins, and oligos labeled by fluorophore used in this study were summarized in Table S1. Probe sequences were designed using OligoMiner software (https://github.com/beliveau-lab/OligoMiner) (87). Combinations of applied probes and branches in the above procedure were summarized in Table S2.

## Data availability

The data that support the findings of this study are available from the corresponding author upon reasonable request.

## Supporting information

This article contains supporting information (38, 39, 88, 89, 90, 91).

## Conflict of interest

The authors declare that they have no conflicts of interest with the content of this article.
