# Supplementary material for: Mammalian type opsin 5 preferentially activates G14 in Gq-type G proteins triggering intracellular calcium response
Source: J Biol Chem. 2023 Jul 7;299(8):105020. doi: 10.1016/j.jbc.2023.105020 (PMC10432815; doi:10.1016/j.jbc.2023.105020)
Supplement: Supporting Information [file mmc1.pdf]

Supporting information for

Mammalian type Opsin 5 preferentially activates G14 in Gq-type G proteins triggering intracellular calcium response

Keita Sato[*,1], Takahiro Yamashita[2], Hideyo Ohuchi[*,1]

[1]*Department of Cytology and Histology, Faculty of Medicine, Dentistry, and Pharmaceutical Sciences, Okayama University*
2-5-1 Shikata-cho, Okayama City, Okayama, 700-8558, Japan
[2]*Department of Biophysics, Graduate School of Science, Kyoto University, Kyoto, 606-8502, Japan*

*Correspondence should be addressed to:
keitasato@okayama-u.ac.jp
ohuchi-hideyo@okayama-u.ac.jp

Supplementary materials and methods

Glosensor cAMP assay

A luminescent cAMP assay using Glosensor 22F as an indicator was performed according to the standard procedure (1). 293T cells were seeded into white-walled 96-well plates (MS-8096W, Sumitomo Bakelite) at 20,000 cells per well in DMEM/F-12 (FUJIFILM Wako) containing 10% FBS. After a day, cells were transfected with 0.05 µg cOpn5L2 expression plasmid and 0.05 µg Glosensor 22F plasmid (Promega) using 0.4 µg polyethyleneimine (PEI) (PEI MAX, Polyscience) diluted in OptiMEM (Thermo Scientific). Six to eight hours after transfection, the medium was replaced with a fresh medium containing 2 µM 11CR. The next day, the medium was replaced with an CO2-independent medium (Thermo Scientific) containing 10% FBS and 2% stock solution of GloSensor cAMP reagent (Promega) under dim red light according to the manufacturer's instructions. After 2 hours of incubation in the dark, luminescence was measured using a microplate luminometer (Veritas, Turner Biosystems). For the increase of cAMP, forskolin was added at final concentration 1 µM. The cells were stimulated with a handheld UV flashlight (0.11± 0.0036 $mW{\cdot}mm^{-2}$; peak irradiance at 373 nm) for 1, 2, or 5 seconds.

Native PAGE, SDS-PAGE, and Western blot

Blue native PAGE and subsequent Western blot were performed to quantify the amount of functionally expressed recombinant G protein alpha subunits. G alpha proteins were transfected into the cells in 24-well plates at 50–60% confluency with polyethyleneimine. A day after transfection, collected cells were solubilized and applied to native polyacrylamide gel, or stored at -80 °C until use. Cell precipitates were solubilized in lysis buffer (1% digitonin, 10 mM 6-aminocaproic acid, 10 mM Bis-Tris, pH 7.0). After centrifugation, supernatants were mixed with 1/4 volume of 5x BN PAGE sample buffer (2.5%(w/v) CBB G-250, 250 mM 6-aminocaproic acid, 50 mM Bis-Tris/HCl, 50%(v/v) glycerol, pH7.0). Cell extracts were separated in 4–10% linear gradient polyacrylamide gel in gel buffer (500 mM 6-aminocaproic acid, 50 mM Bis-Tris, pH7.0) with anode (50 mM Bis-Tris, pH 7.0) and cathode (50 mM Tricine, 15 mM Bis-Tris, 0.02% CBB G-250, pH 7.0) buffers. ExcelBand enhanced 3-color regular range protein marker (PM2510, SMOBIO) and EzStandard Native (WSE-7016, ATTO) were also applied as molecular weight markers. Electrophoresis was performed at 100 V for 1 hour, and subsequent 3–4 hours run with 150V or overnight run with 1 mA. The cathode buffer was replaced with the same buffer with CBB G-250 reduced to 0.002% after 2 hours from start. The gels were chilled to 6-8 °C during electrophoresis. The electrophoresis was stopped when the thick blue line of the CBB G-250 approached the gel front. The gel was soaked in the SDS running buffer (0.1% (w/v) SDS, 25 mM Tris, 192 mM glycine) at RT for 30 minutes

to denature the proteins in the gel. Denatured proteins were transferred onto PVDF membrane with blotting buffer (25 mM Tris, 192 mM glycine, 5% (v/v) methanol) in tank blotter (Criterion, BioRad) at a constant voltage 65 V for 45 minutes. After removal of CBB G-250 with methanol, PVDF membranes were stained with ponceau S and marked at molecular weight markers with luminopen. After removal of ponceau S with TBST buffer (50 mM Tris/HCl, 150 mM NaCl, 0.1% Tween-20, pH 7.5) and blocking with Blocking One (Nacalai Tesque), PVDF membranes were incubated with respective antibodies overnight at 4 °C, washed with TBST buffer three times for 15 minutes, and incubated with corresponding peroxidase-conjugated secondary antibodies for 1 hour at RT. Primary and secondary antibodies were diluted with IMMUNO SHOT 1 and 2 (Cosmo bio), respectively. After three times wash with TBST, chemiluminescent images were taken with ImmunoStar LD (FUJIFILM WAKO) as luminescence substrate by C-DiGit chemical luminescence scanner (LI-COR). For SDS-PAGE, the sample was extracted with RIPA buffer containing 0.1% SDS (Nacalai Tesque). SDS-PAGE was run in the 12.5 % polyacrylamide running gels with SDS running buffer. Protein transfer to PVDF membrane, application of antibodies, and chemiluminescent detection were performed as same as done after blue native PAGE except for removal of CBB G-250 and staining with ponceau S. Image analysis was performed with Image Studio Lite Ver 5.2 and Image Studio Digits Ver 4.0 (LI-COR). Used antibodies and dilutions were listed in the Table S3.

Quantitative PCR

Total RNA extracted from medaka tissues by Direct-zol RNA Microprep Kits (R2063; Zymo Research), contaminant genomic DNA removal by AccuRT Genomic DNA Removal Kit (Applied Biological Materials), and first strand cDNA synthesis by FastGene cDNA Synthesis 5x ReadyMix (NE-LS64; Nippon genetics) were carried out according to manufacturers' instructions. Quantitative PCR was performed with Light Cycler Nano (Roche Diagnostics) using THUNDERBIRD Next SYBR qPCR Mix (QPX-201; Toyobo) according to the manufacturer's instructions. The relative expression levels of the target genes were estimated by the comparative threshold cycle method, normalized by the internal control (*rpl7*) as ΔCq. Primer sequences used in quantitative PCR are listed in Table S1.

In situ hybridization

Chromogenic in situ hybridization was performed according to Sato et al. 2021 (2).

Supplementary figures

```
                    10        20        30        40        50        60        70        80        90       100
           ....|....|....|....|....|....|....|....|....|....|....|....|....|....|....|....|....|....|....|....|
Human Rho  MNGTEGPNFYVPFSNATGVVRSPFEYPQY------YLAEPWQFSMLAAYMFLLI--VLGFPINFLTLYVTVQHKKLRTPLNYILLNLAVADLFMVLGGFT
hOPN5      M------------ALNHTALPQDERLPHYLRDGDPFASKLSWEADLVAGFYLTIIGILSTFGNGYVLYMSSRRKKKLRPAEIMTINLAVCDLGISVVGKP
mOpn5      M------------ALNHTALPQDERLPHYLRDEDPFASKLSWEADLVAGFYLTIIGILSTFGNGYVLYMSSRRKKKLRPAEIMTINLAVCDLGISVVGKP
cOpn5m     MSGM---------ASDCNSSSQEEYLPHYVQQEDPFASKLSREADIIAGFYLTVIGILSTLGNGYVIFMSSKRKKKLRPAEIMTVNLAVCDLGISVVGKP
XtOpn5m    M------------AGNSSYREESGYIPHYERDSDPFASKLSREADIFAGVYLMAIGILSTLGNGYVIYMACSRKKKLRPAEIMTINLAVCDLGISVTGKP
DrOpn5m    M--------------ENETSISSGYIPHYLLRGDPFASKLSKEADIVAAFYILVIGILSATGNGYVMYMTFKRKTKLKPPEIMTLNLAIFDFGISVSGKP
OlOpn5m    M--------------ENTSWPHSSYVPHYLLRGDPFASRLSKEADIVAAFYILIIGILSATGNGYVIYMTIKRKTKLKPPELMTVNLAIFDFGISVTGKP
DrOpn5m2   MSKRVN-------GSWMGSVSVPSSIPH------AFRSQLSPSADLSIAVFLIITGVVSVFGNGLVLLVYGQRRKKLRAHELMTINLAVCDFGYSLLGAP
cOpn5L2    M-------------------------------EEQYISKLHPVVDYGAGVFLLIIAILTILGNSAVLATAVKRSSLLKSPELLTVNLAVADIGMAISMYP
                   110       120       130       140       150       160       170       180       190       200
           ....|....|....|....|....|....|....|....|....|....|....|....|....|....|....|....|....|....|....|
Human Rho  STLYTSLHGYFVFGPTGCNLEGFFATLGGEIALWSLVVLAIERYVVVCKPMS-NFRFGENHAIMGVAFTWVMALACAAPPLAGWSRYIPEGLQCSCGIDY
hOPN5      FTIISCFCHRWVFGWIGCRWYGWAGFFFGCGSLITMTAVSLDRYLKICYLSY-GVWLKRKHAYICLAAIWAYASFWTTMPLVGLGDYVPEPFGTSCTLDW
mOpn5      FTIISCFCHRWVFGWFGCRWYGWAGFFFGCGSLITMTAVSLDRYLKICYLSY-GVWLKRKHAYICLAVIWAYASFWTTMPLVGLGDYAPEPFGTSCTLDW
cOpn5m     FSIISFFSHRWIFGWMGCRWYGWAGFFFGCGSLITMTAVSLDRYLKICHLAY-GTWLKRHHAFICLALIWAYATFWATVPFAGVGSYAPEPFGTSCTLDW
XtOpn5m    FAIVSCFSHRWVFGWNACRWYGWAGFFFGCGSLITLTVVSLDRYLKICHLRY-GTWLKRRHAFIALAVIWAYATLWATLPLVGVGNYAPEPFGTTCTLDW
DrOpn5m    FFIVSSFSHRWLFGWQGCRYYGWAGFFFGCGSLITMTVVSFDRYLKICHLRY-GTWLKRHHAFLSVVFIWAYAAFWATMPVVGWGNYAPEPFGTSCTLDW
OlOpn5m    FFVVSSFSHRWLFGWEGCRFYGWAGFFFGCGSLITMTVVSLDRYLKICHLRY-GTWLKRQHIFLCLVFVWIYAAFWATMPLVGWGSYAPEPFGTSCTLDW
DrOpn5m2   CIIISSLSHAWIFGETGCIWYGIQGFVFGIGSLITTCLISLDRCFKICSFRY-GQWVERKHASLSVTLVWIYTLFWASLPMFGFGSYGPEPFGTSCTINW
cOpn5L2    LAIASAWNHAWLGGDASCIYYALMGFLFGVCSMMTLCAMAVIRFLVTNSSKSNSNKISKNTVHILITFIWLYSLLWAILPLVGWGYYGPEPFGISCTIAW
                   210       220       230       240       250       260       270       280       290       300
           ....|....|....|....|....|....|....|....|....|....|....|....|....|....|....|....|....|....|....|
Human Rho  YTLKPEVNNESFVIYMFVVHFTIPMIIIFFCYGQLVFTVKEA----AAQQQESATTQKAEKEVTRMVIIMVIAFLICWVPYASVAFYIFTHQGSN-----
hOPN5      WLAQASVGGQVFILNILFFCLLLPTAVIVFSYVKIIAKVKSSSKEVAHFDSRIHSSHVLEMKLTKVAMLICAGFLIAWIPYAVVSVWSAFGRPDS-----
mOpn5      WLAQASGGGQVFILSILFFCLLLPTAVIVFSYAKIIAKVKSSSKEVAHFDSRIHSSHVLEVKLTKVAMLICAGFLIAWIPYAVVSVWSAFGRPDS-----
cOpn5m     WLAQASVAGQAFVLSILFFCLLFPTAVIVFSYVKIILKVKSSTKEVAHYDTRIQNSHILEMKLTKVAMLICAGFLIAWIPYAVVSVWSAFGQPDS-----
XtOpn5m    WLAQASVKGQIFVLSMLFFCLLFPTMVIVFSYAKIIAKVKSSAKEVAHFDTRNQNNHTLEIKLTKVAMLICAGFLIAWFPYAVVSVWSAFGQPDS-----
DrOpn5m    WLTQASVSGQSFVMCMLFFCLIFPTVIIVFSYVMIIFKVKSSAKEVSHFDTRNKNNHSLEMKLTKVAMLICAGFLIAWIPYAVVSVMSAFGEPDS-----
OlOpn5m    WLAQASVSGQSFVMSILFFCLVLPAGIIVFSYVMIICKVKSSSKEVSSFDARIKNSHTLEIKLTKVAMLICAGFLIAWIPYAVVSVVSAFGEPDS-----
DrOpn5m2   WRMNSSLTDRIYIFLILSLCFGVPTLIIIASYLAIVIRVYWSSHILASIPSSNVTHNSKDLRLTKIAAVVCSSFLIAWTPYAIVSLYSAVMAREEQLGED
cOpn5L2    SKFHSSSNGFSFILSMFLLCTVLPALTIVACYLGIAWKVHKAYQEIQNIN-RIPHAAKLEKKLTLMAVLISVGFLSAWTPYAAASFWSIFNSSDS-----
                   310       320       330       340       350       360       370       380       390       400
           ....|....|....|....|....|....|....|....|....|....|....|....|....|....|....|....|....|....|....|
Human Rho  --------------------------------------------------------------------------------FGPIFMTIPAFFAKSAAIYN
hOPN5      --------------------------------------------------------------------------------IPIQLSVVPTLLAKSAAMYN
mOpn5      --------------------------------------------------------------------------------IPIQLSVVPTLLAKSAAMYN
cOpn5m     --------------------------------------------------------------------------------VPIQFSVVPTLLAKSAAMYN
XtOpn5m    --------------------------------------------------------------------------------IPIELSVVPTMMAKSASMYN
DrOpn5m    --------------------------------------------------------------------------------VPIPVSVVPTLLAKSSAMYN
OlOpn5m    --------------------------------------------------------------------------------IPIPVSVIPTLLAKSSAMYN
DrOpn5m2   GGTAIPGGITGTGIGRGGGFPDVYAFPFLINWTSSEQFGDVFGSWRNMTSGHSHSVGHHGSNVGSGHGGTGGHHSLPESILKPEFSLIPAIFAKSHCMIN
cOpn5L2    --------------------------------------------------------------------------------LQPIVTLLPCLFAKSSTAYN
                   410       420       430       440       450       460       470       480       490       500
           ....|....|....|....|....|....|....|....|....|....|....|....|....|....|....|....|....|....|....|
Human Rho  PVIYIMMNKQFRNCMLTTICCGKNPLGDDEASATVSKT-------------------ETSQVA----PA-------------------------------
hOPN5      PIIYQVIDYKF-------ACCQTGGLKATKKK-SLEGF----------RLHTVTTVRKSS-------AVLEIHEEWE-----------------------
mOpn5      PIIYQVIDYRF-------ACCQAGGLRGTKKK-SLEDF----------RLHTVTAVRKSS-------AVLEIHPESS-----SRFTSAHVMDG-------
cOpn5m     PIIYQVIDCKF-------ACCRSGGPKTLQKKSSLKES----------RMYTLSSHRDSA-------ALSGTQLEV------------------------
XtOpn5m    PIIYQVIDCKP-------ACCK-------KDK-SLQNT--------TSRVYTISTFRKST-------TSAR-----------------------------
DrOpn5m    PIIYQVIDCKK-------KCVKSCCFQAWRKKKPSKTS----------RFYTISGSIKQR-------PADEASIEI------------------------
OlOpn5m    PIIYQLVDLKN-------SC-STCCAKVIRKRTHFRNS----------RFYTISGSLKDTA------PAKEAHIEI------------------------
DrOpn5m2   PFIYQIMNRDFREDVYDLLCMR-GKDGERRRSRSTDGSDSDGHRGSVTLSYIHSWRRRST---PTNQPSEK--CVKKESSRKSRGKRGSCQDNNSIGWAT
cOpn5L2    PFIYYIFSKTFRHEIKQLQCCWGWRVHFFSADNSAENS--------VSMMWSGRDNIRLS-------PTAK--VESQGAARH------------------
                   510       520       530       540
           ....|....|....|....|....|....|....|....|.
Human Rho  -----------------------------------------
hOPN5      -----------------------------------------
mOpn5      -----------------------------ESHSNDGDCGKK
cOpn5m     -----------------------------------------
XtOpn5m    -----------------------------------------
DrOpn5m    -----------------------------------------
OlOpn5m    -----------------------------------------
DrOpn5m2   LDTTTLDTQVNLEREKGKEVEIETQKVFKESSSSSLLTVKE
cOpn5L2    -----------------------------------------
```

Supplementary figure 1. Multiple alignment of amino acid sequences of opsins investigated in this study.

Alignment of the amino acid sequences was performed by MAFFT (3). Cytoplasmic loops and C-terminal regions are highlighted in yellow. N-terminal regions and extracellular loops are highlighted in cyan. The accession numbers of the amino acid sequences were as follows: human Rhodopsin (Human Rho), NP_000530; human OPN5 (hOPN5), NP_859528; mouse Opn5 (mOpn5), NP_861418; chicken Opn5m (cOpn5m), NP_001124215; *Xenopus tropicalis* Opn5m (XtOpn5m), XP_002936036; zebrafish Opn5m (DrOpn5m), NP_001186975; medaka Opn5m (OlOpn5m), XP_023808946; zebrafish Opn5m2 (DrOpn5m2), NP_001304680; chicken Opn5L2 (cOpn5L2), NP_001156364.

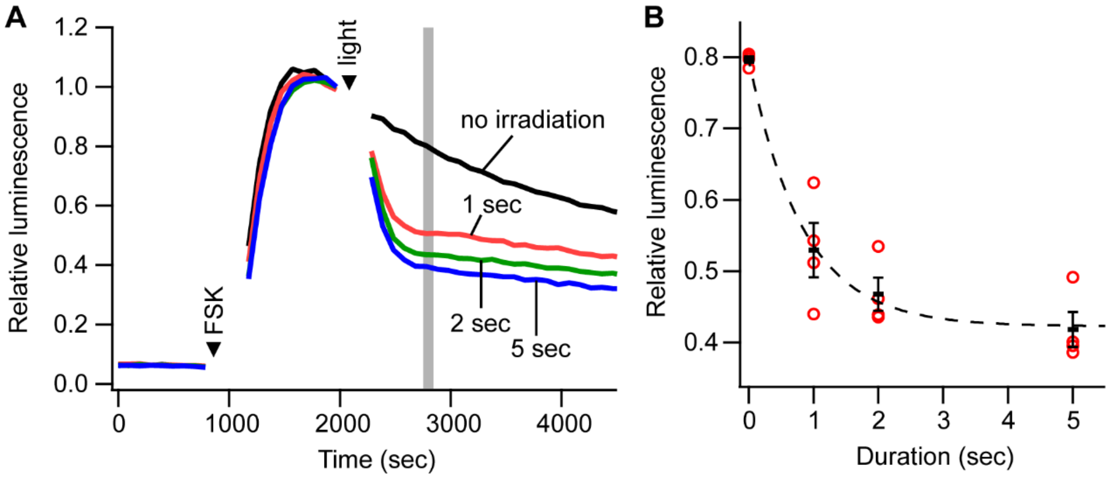

Supplementary figure 2. Photosensitivity evaluation of cOpn5L2 by cAMP bioluminescence assay.

To examine whether the UV light conditions in the aequorin assay were sufficient to activate cOpn5L2 and subsequent downstream signaling cascade, changes in intracellular cAMP levels were monitored using the bioluminescent cAMP sensor Glosensor 22F under varying durations of UV light exposure (1, 2, and 5 sec). (A) Representative traces of relative luminescence change in Glosensor 22F by addition of forskolin (FSK) and light activation of cOpn5L2. Luminescence intensity is normalized to that immediately before light irradiation. Final concentration of FSK was 1 μM. (B) Relative luminescence values at 800 sec after light irradiation (2,800 sec after start of recording, gray vertical line in panel A) were plotted against duration of UV light irradiation. Red circles and error bars indicate individual data and standard error of the mean (s.e.m.), respectively. The broken curve is the exponential fitting of the data (0.423+0.373*exp(-t/0.838)).

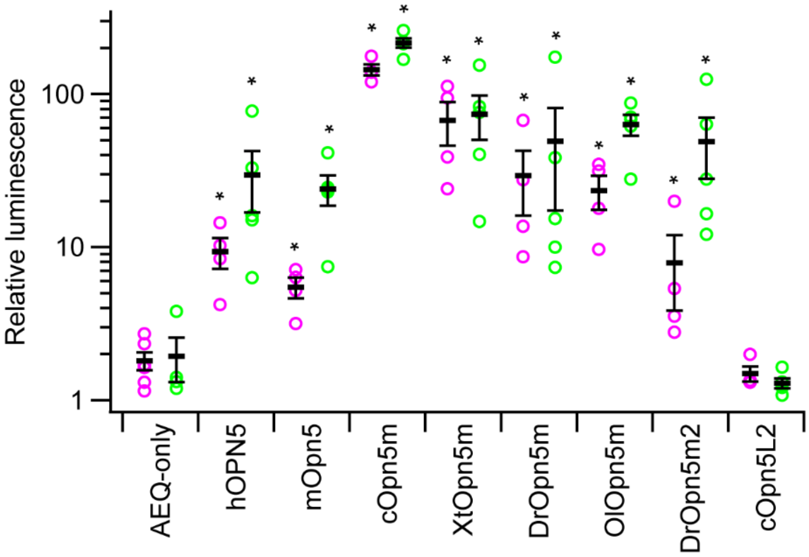

Supplementary figure 3. Calcium response in WT 293T by Opn5m proteins supplied with ATR and 11CR.

Intracellular calcium response investigated by aequorin luminescence assay in WT 293T cells. After measurement of aequorin luminescence before and after UV light irradiation, relative luminescence values were calculated by dividing the luminescence intensity values just after irradiation by those immediately before irradiation. Magenta and green circles show the individual data from ATR- and 11CR-reconstituted samples, respectively. Error bars show s.e.m. Dunnett's test was used for comparison to aequorin (AEQ)-only transfection (*$P<0.05$). The data for ATR-reconstituted samples are identical to those shown in Figure 2B of the main text. Source raw luminescence traces were summarized in Supplementary figure 13.

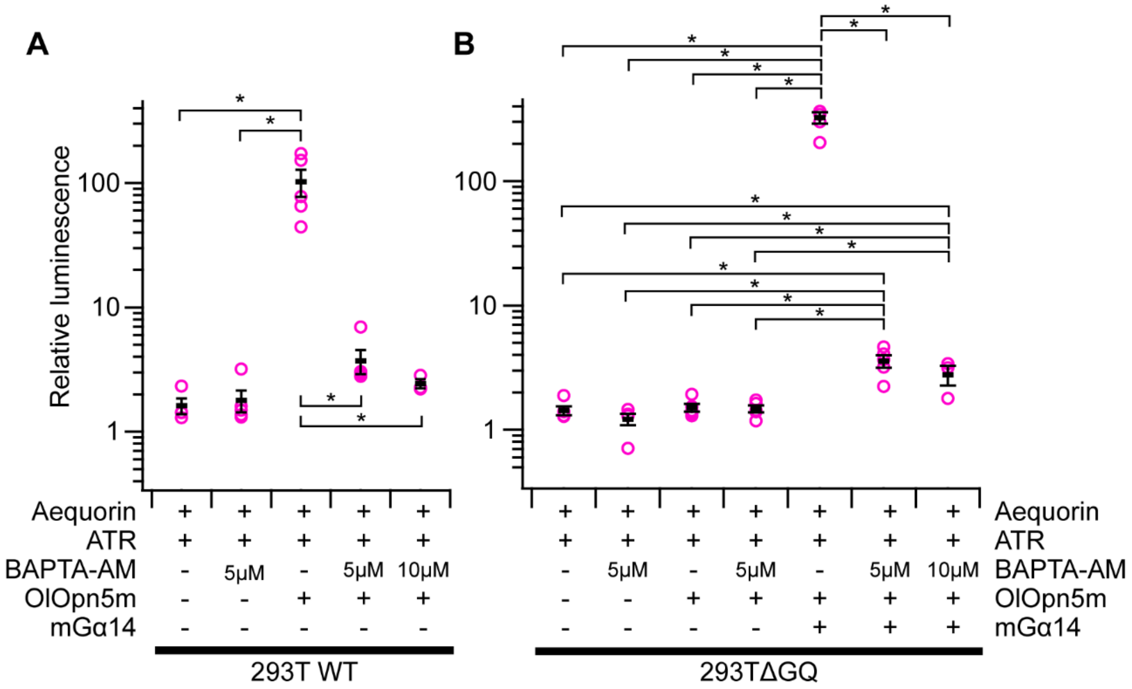

Supplementary figure 4. Cytosolic calcium chelator abolishes luminescent increase of aequorin by Opn5m.

(A) Aequorin assay in 293T WT cell line. When OlOpn5m was introduced in the presence of 5 μM ATR, relative luminescence increased, and this was abolished by cytosolic calcium chelator BAPTA-AM. BAPTA-AM was applied at 5 μM or 10 μM when the culture medium was replaced with L-15 containing coelenterazine h. (B) Aequorin assay in 293TΔGQ cells. Relative luminescence increased when both OlOpn5m and mGα14 was introduced in the presence of 5 μM ATR, which was abolished by BAPTA-AM applied at 5 μM or 10 μM. ATR alone does not increase the luminescence, which is not altered by BAPTA-AM. OlOpn5m alone does not increase the luminescence, which is not altered by BAPTA-AM. Relative luminescence values were calculated by dividing the luminescence intensities immediately after irradiation by those before irradiation. Individual data points are shown in magenta. Error bars show s.e.m. The Tukey-Kramer test was used for multiple comparisons and no significant difference was identified except for indicated pairs (*P<0.05). Source raw luminescence traces were summarized in Supplementary figure 13.

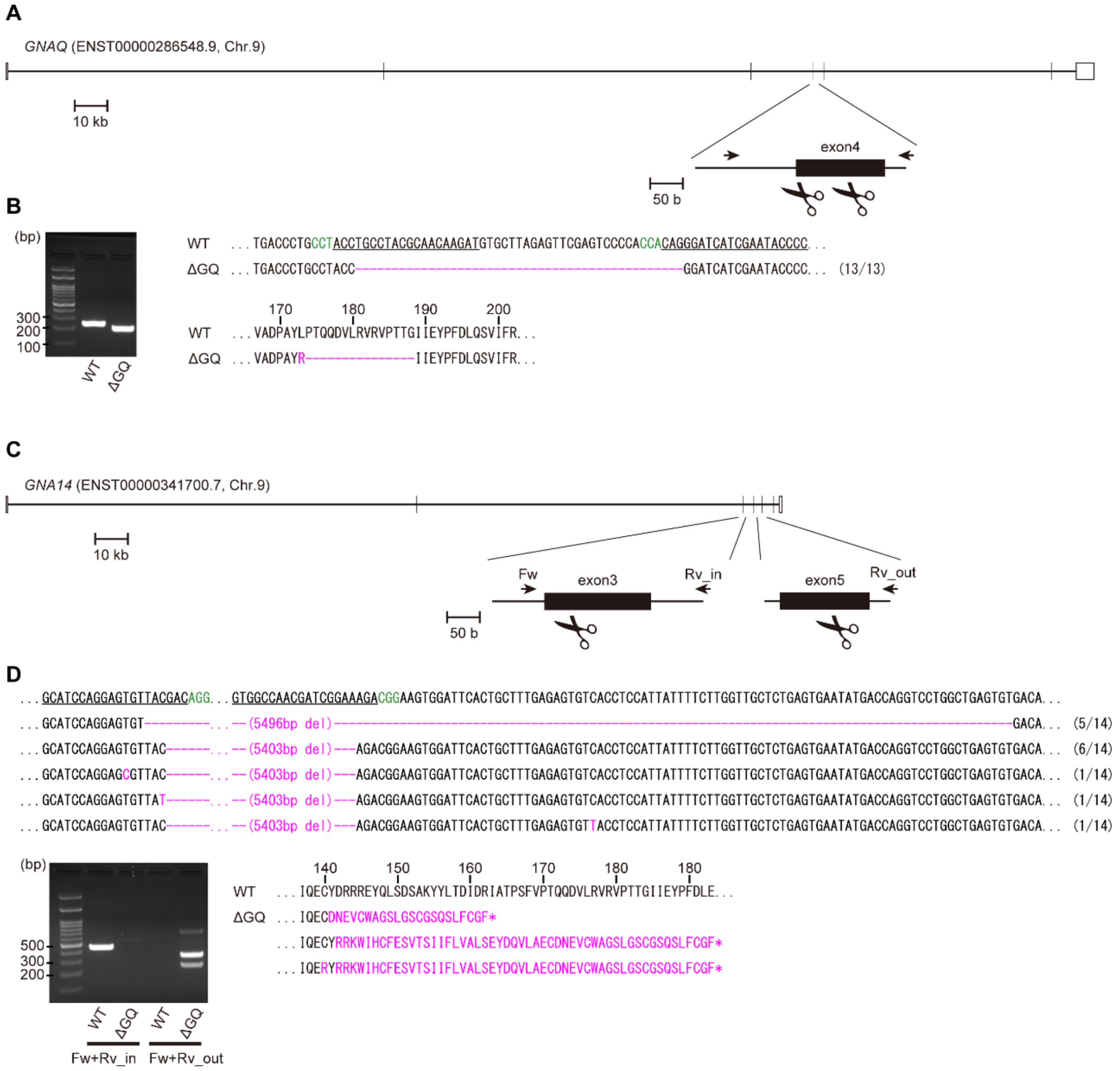

(Supplementary figure 5, continued)

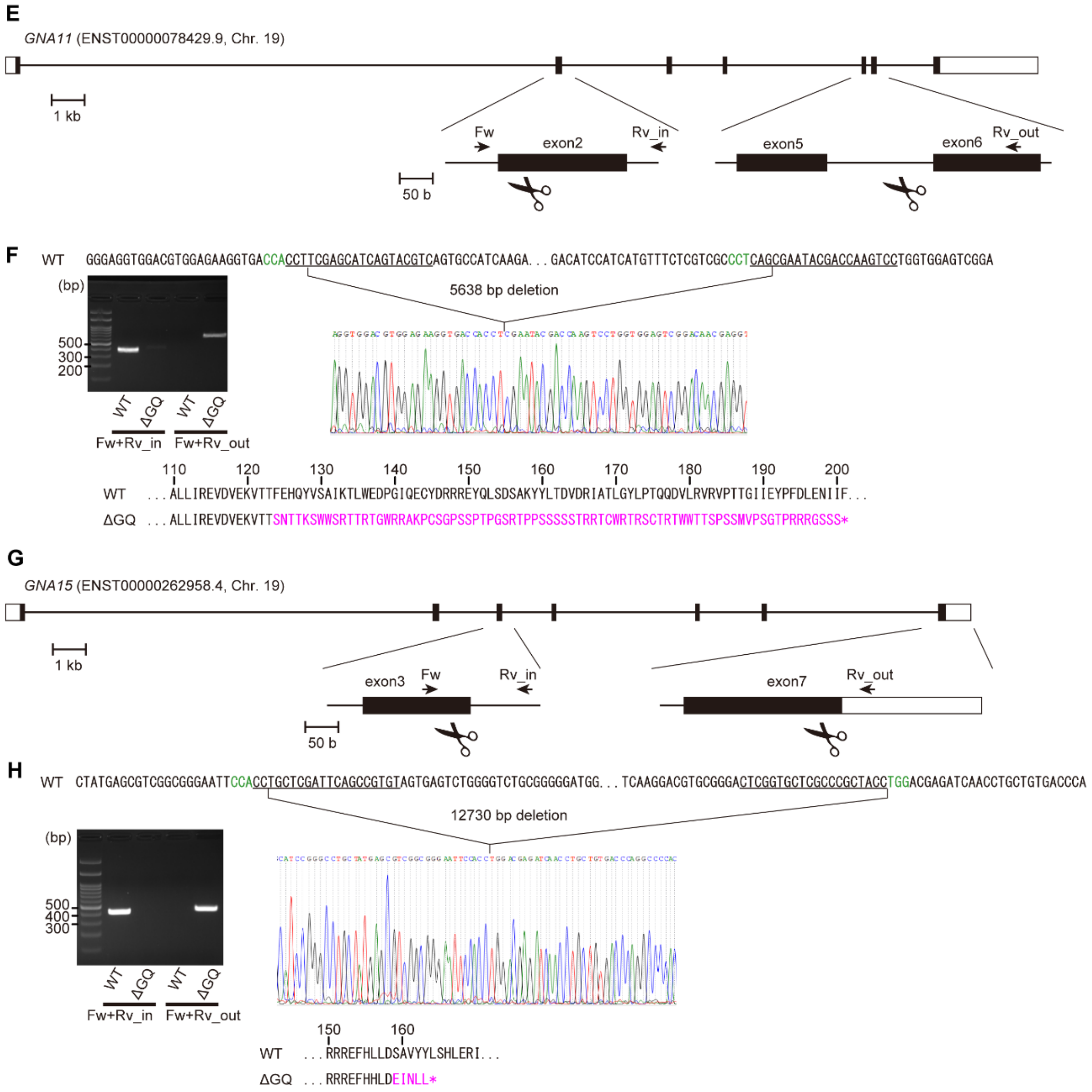

Supplementary figure 5. Targeted sequences for genome editing and resultant genotype of 293TΔGQ produced in this study.

To confirm the edited sequence of the 293T genome, genomic PCR products were directly sequenced in the case of *GNA11* and *GNA15*. For *GNAQ* and *GNA14*, genomic PCR products were ligated into T-Vectors pMD20 (Takara) and transformed into *E. coli*. Colony directed PCR was then performed from several clones and sequenced. In the schematic drawings of gene structures, filled and open boxes indicate the coding and untranslated regions in exons, respectively. Arrows and scissors indicate the positions of primers for genotyping and those of the target sequences for SpCas9 cleavage, respectively. Green letters and underlines in nucleotide sequences are protospacer adjacent motifs (PAM) and the sequences targeted by sgRNA, respectively. Magenta letters in nucleotide and amino acid sequences indicate the deletion or replacement in the mutant sequence.

(A) Gene structure, sgRNA-targeted sites, and primer binding sites of *GNAQ*.
(B) The gel electrophoresis result of genomic PCR and sequences determined from cloned colonies. Thirteen clones were sequenced, and all of them showed 45 bp deletion. Deduced amino acid sequence is shown below. Deletion of the amino acid sequence (from L173 to G188) removes functionally essential magnesium binding site (T186) and G2 motif (Switch I, 178–186).
(C) Gene structure, sgRNA-targeted sites, and primer binding sites of *GNA14*.
(D) The gel electrophoresis result of genomic PCR and sequences determined from cloned colonies. The genomic PCR was performed by two primer pairs Fw/Rv_in and Fw/Rv_out. The binding site for the primer Rv_in is removed in ΔGQ. The binding site for the primer Rv_out is much distant (approximately 5.5 kb) from the primer Fw for amplification in WT in this experimental condition. Among 14 sequenced clones, 5 and 9 of them showed 5496 and 5403 bp deletion, respectively. In 9 clones with deletion of 5403 bp, three clones showed an additional single nucleotide mutation at three sites each. The amino acid sequences deduced from the mutated nucleotide sequences are C-terminally truncated by frameshift.
(E) Gene structure, sgRNA-targeted sites, and primer binding sites of *GNA11*.
(F) The gel electrophoresis and the direct sequencing of genomic PCR. The genomic PCR was performed by two primer pairs Fw/Rv_in and Fw/Rv_out. The PCR product amplified by Fw/Rv_out from genomic DNA of 293TΔGQ was sequenced. The sequencing result shows the deletion of 5638 bp. The amino acid sequence deduced from the mutated nucleotide sequences is C-terminally truncated.
(G) Gene structure, sgRNA-targeted sites, and primer binding sites of *GNA15.*
(H) The gel electrophoresis and the direct sequencing of genomic PCR. The genomic PCR was performed by two primer pairs Fw/Rv_in and Fw/Rv_out. The PCR product amplified by Fw/Rv_out from genomic DNA of 293TΔGQ was sequenced. The sequencing result shows the deletion of 12730 bp. The amino acid sequence deduced from the mutated nucleotide sequences is C-terminally truncated.

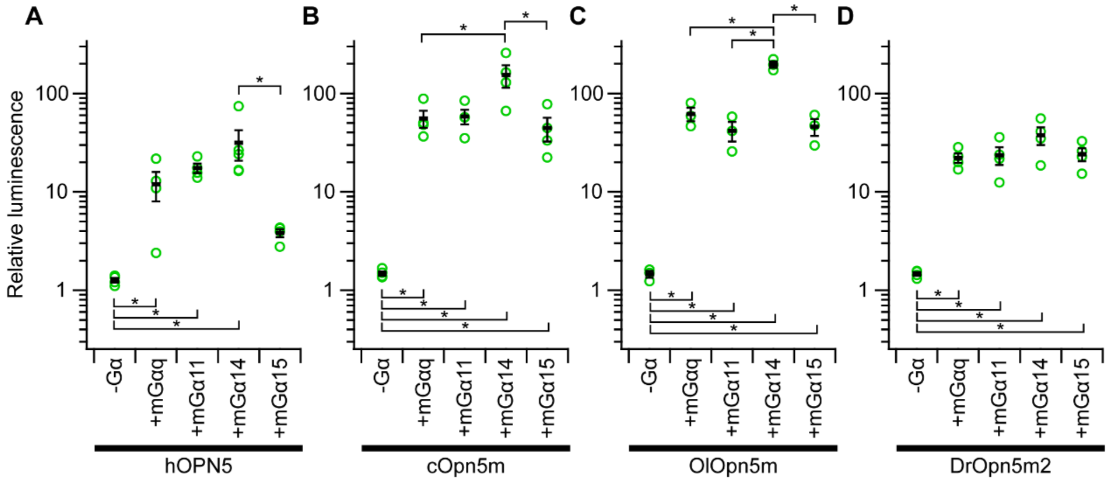

Supplementary figure 6. Calcium response in 293TΔGQ cells by Opn5m proteins supplied with 11CR.

Intracellular calcium response of hOPN5 (A), cOpn5m (B), OlOpn5m (C), and DrOpn5m2 (D) investigated by aequorin luminescence assay in 293TΔGQ cells. After measurement of aequorin luminescence before and after UV light irradiation, relative luminescence values were calculated by dividing the luminescence intensity values just after irradiation by those immediately before irradiation. Green circles show the individual data from 11CR-reconstituted samples. Error bars show s.e.m. The Tukey-Kramer test was used for multiple comparisons (*$P<0.05$). Source raw luminescence traces are summarized in Supplementary figure 13.

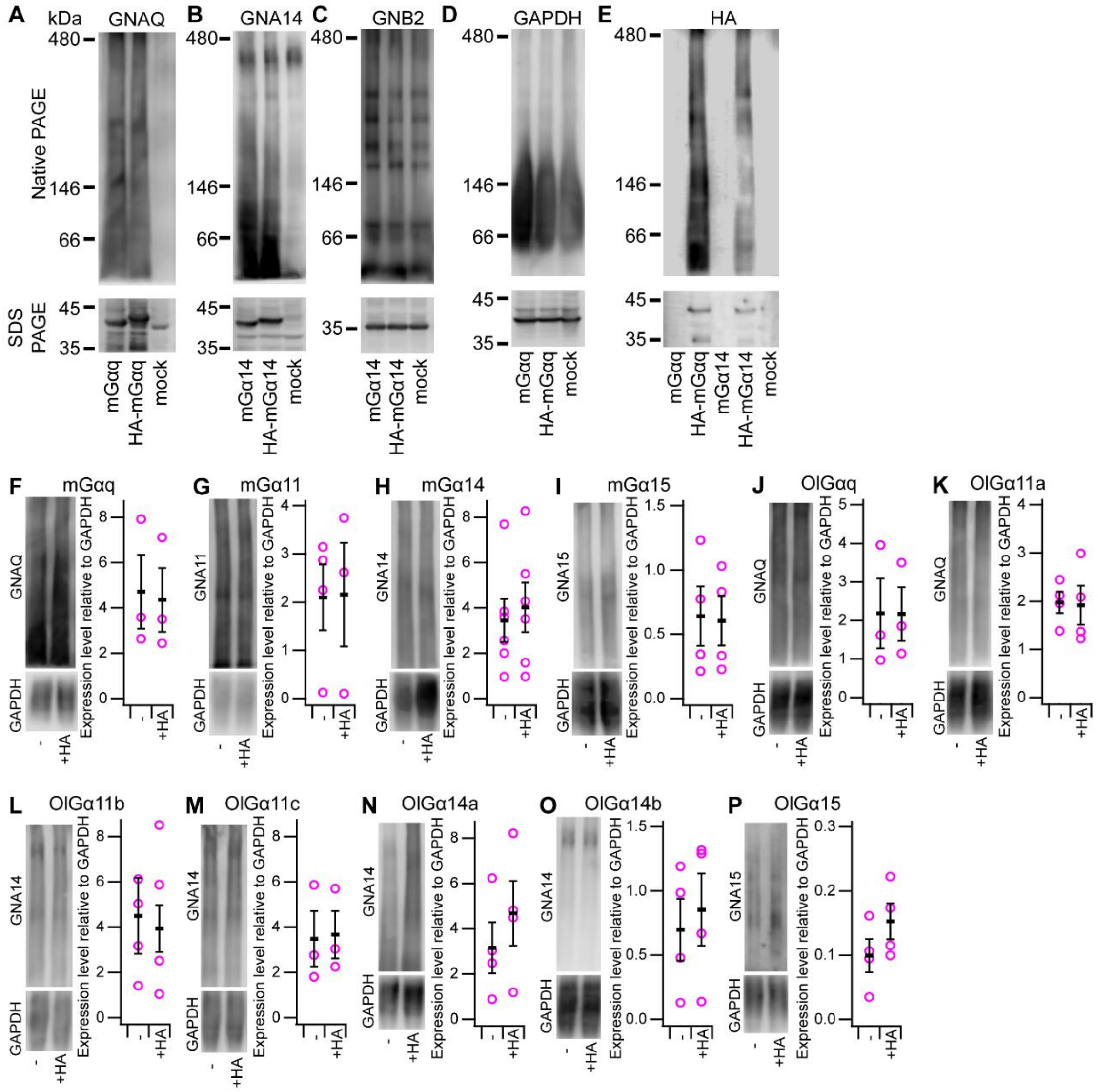

(Supplementary figure 7, continued)

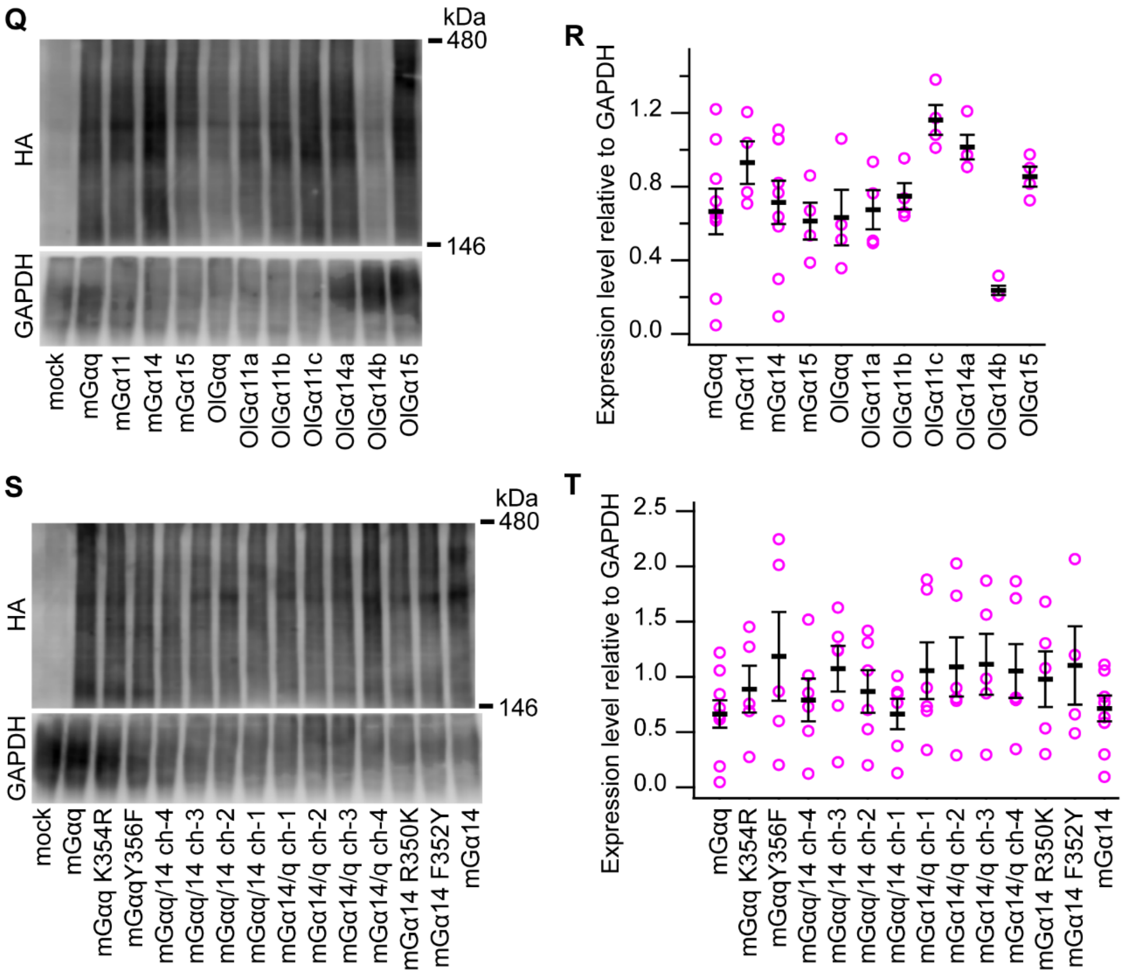

Supplementary figure 7. Estimation of expression level of recombinant Gα capable of making protein complex.

Expression levels of functional recombinant alpha subunits of G proteins in this study were estimated by blue native PAGE and subsequent Western blot.

(A-E) Representative Western blotting images after blue-native PAGE and SDS-PAGE. 293TΔGQ cells transfected with recombinant mGαq, HA-tagged mGαq, mGα14, and HA-tagged mGα14 in addition to mock-transfected cells were lysed with lysis buffer containing 1% digitonin for native PAGE or RIPA buffer containing 0.1% SDS for SDS-PAGE. Western blot experiments with anti-GNAQ (A), anti-GNA14 (B), and anti-GNB2 (C) show much larger molecular weight product in native PAGE than in SDS-PAGE. Majority of GNB2 forms probable multi-protein complexes seen between 146 kDa and 480 kDa molecular weight markers in native PAGE, although those identities are not clear (C). According to previous reports, it is most likely that they include heterotrimeric G protein pre-coupled with GPCR (4, 5). While overexpressed mGαq and mGα14 showed smeared signals compared to the endogenous GNB2 possibly due to heterogeneity in protein folding and lipid modification,

they also showed the signals in the region between 146 kDa and 480 kDa molecular weight (A, B). Loading control GAPDH was mainly detected between 146 kDa and 66 kDa markers (D). HA-tagged Gα was detected in native PAGE as well as in SDS-PAGE with anti-HA antibody (E). According to these data, a relative amount of functional recombinant Gα was estimated as Gα-containing complexes between 146 kDa and 480 kDa molecular weight normalized to endogenous GAPDH level.

In panels F-T, the blotted PVDF membrane was separated at 146 kDa marker to upper and lower fragments for detection of Gα-containing complex and GAPDH, respectively. (F-P) Comparison of expression levels of untagged and HA-tagged Gα proteins. mGαq (F), OlGαq (J), OlGα11a (K), and those tagged with HA were detected by anti-GNAQ. mGα11 and HA-tagged mGα11 were detected by anti-GNA11 (G). mGα14 (H), OlGα11b (L), OlGα11c (M), OlGα14a (N), OlGα14b (O), and those tagged with HA were detected by anti-GNA14. mGα15 (I), OlGα15 (P), and those tagged with HA were detected by anti-GNA15. Representative images of Western blot of Gα and corresponding GAPDH were shown. Densitometric values derived from Gα-containing complexes were normalized by those of GAPDH. Individual data are shown in magenta. Error bars show s.e.m. This result showed that HA-tag insertions summarized in Supplementary figure 9B did not compromise the expression level and protein folding of Gα.

(Q-T) Quantification of HA-tagged WT Gα (Q, R) and HA-tagged chimeric and point mutants of mGαq and mGα14 (S, T). Panels Q and S show representative blot images detected by anti-HA and anti-GAPDH. Panels R and T show densitometric values derived from complexes containing HA-tagged Gα were normalized by those of GAPDH as a loading control. Individual data are shown in magenta. Error bars show s.e.m.

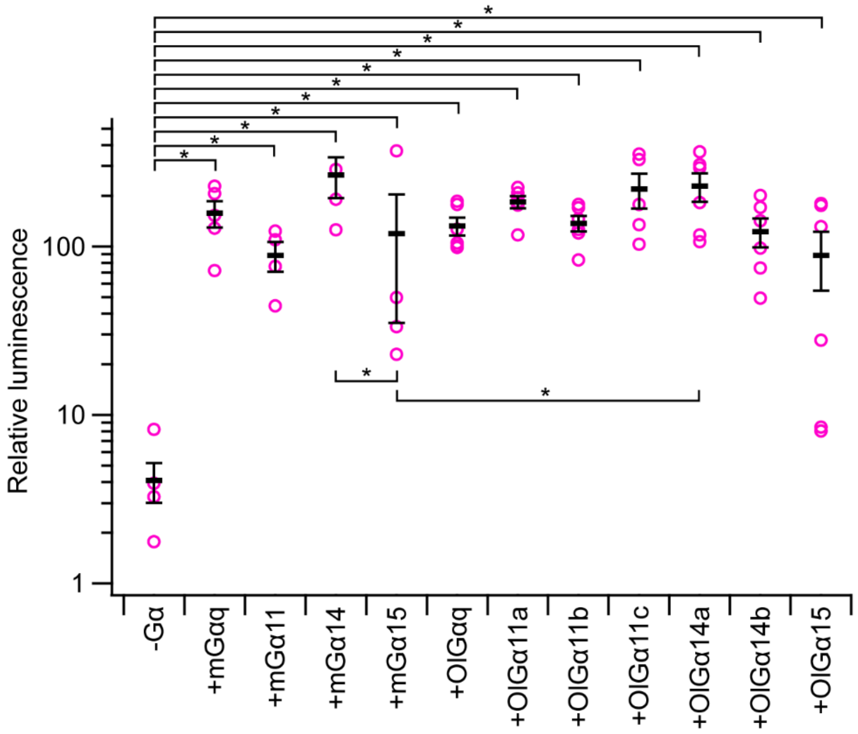

Supplementary figure 8. Activation of Gq-type Gα proteins by jumping spider rhodopsin (JSR).

An aequorin assay was performed in 293TΔGQ cell line with JSR and Gq-type Gα proteins from mouse and medaka. The culture medium was supplemented with 5 μM ATR for reconstitution of JSR. Light irradiation was performed for five seconds with blue LED (~1.7 mW·mm^{-2}; peak irradiance at 466 nm). Relative luminescence values were calculated by dividing the luminescence intensities just after irradiation by those immediately before irradiation. Individual data points are shown in magenta. Error bars show s.e.m. The Tukey-Kramer test was used for multiple comparisons (*P<0.05). The results show that every Gq protein examined in this assay significantly and comparably increases luminescence in JSR-transfected 293TΔGQ cells. Source raw luminescence traces are summarized in Supplementary figure 13.

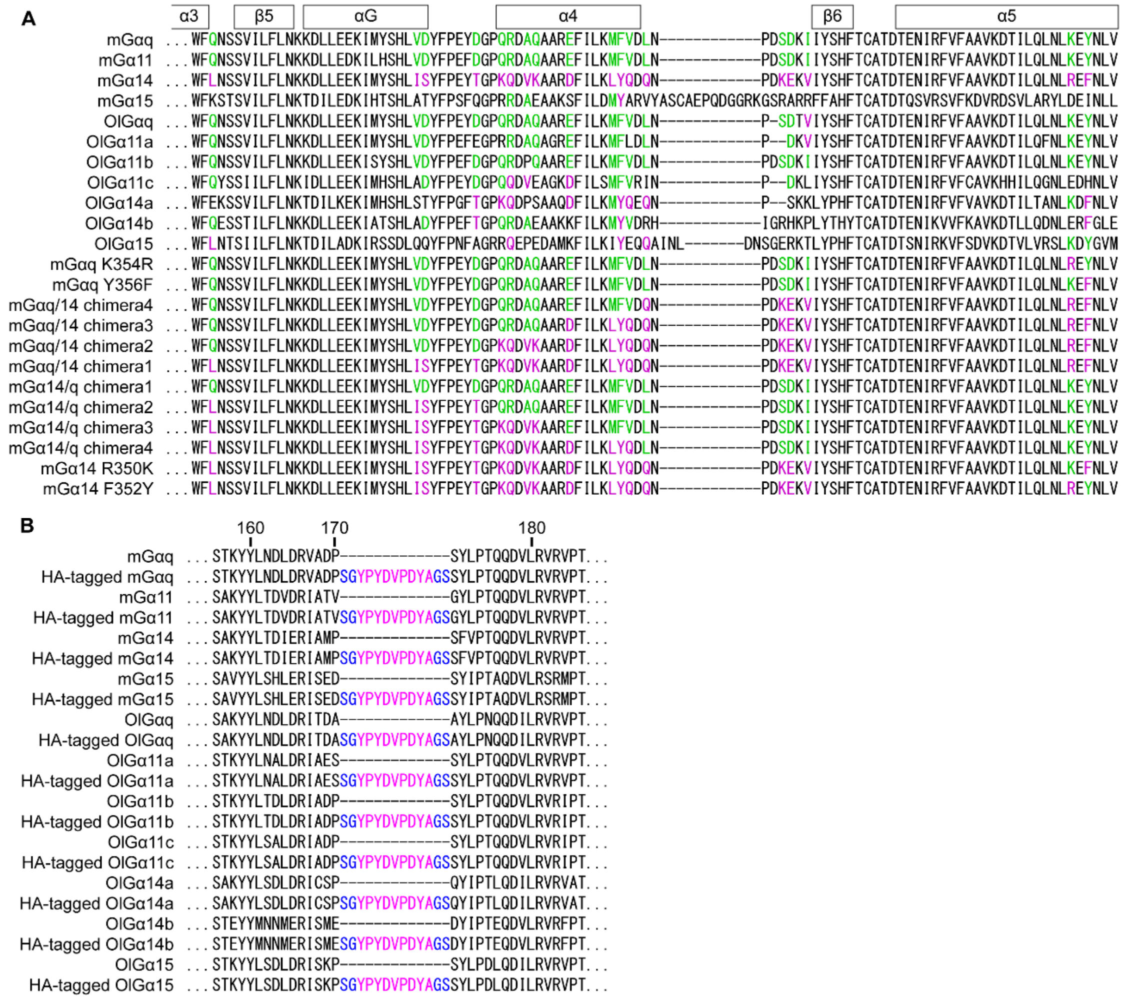

Supplementary figure 9. Alignment of amino acid sequences of Gα proteins.

(A) Amino acid sequence alignment of C-terminal side of Gα proteins tested in this study. Alignment was performed by MAFFT (3). Amino acids that differ between mGαq and mGα14 are highlighted in green and magenta and those for medaka Gα proteins are similarly highlighted. Secondary structural features of mGαq are shown above. The accession numbers of the amino acid sequences were as follows: mouse Gαq (mGαq), NP_032165; mouse Gα11 (mGα11), NP_034431; mouse Gα14 (mGα14), NP_032163; mouse Gα15 (mGα15), NP_034434; medaka Gαq (OlGαq), XP_023814176; medaka Gα11a (OlGα11a), XP_004068399; medaka Gα11b (OlGα11b), XP_020569601; medaka Gα11c (OlGα11c), XP_023816641; medaka Gα14a (OlGα14a), XP_011477393; medaka Gα14b (OlGα14b), XP_004074607; medaka Gα15 (OlGα15) XP_023810335. (B) Amino acid sequence alignment to indicate where HA-tag is inserted. These HA-tagged Gα proteins were used to quantify functional recombinant Gα proteins shown in Supplementary figure 7. The position of HA tag insertion was determined according to the previous reports (6). HA tag and linkers are highlighted in magenta and blue, respectively.

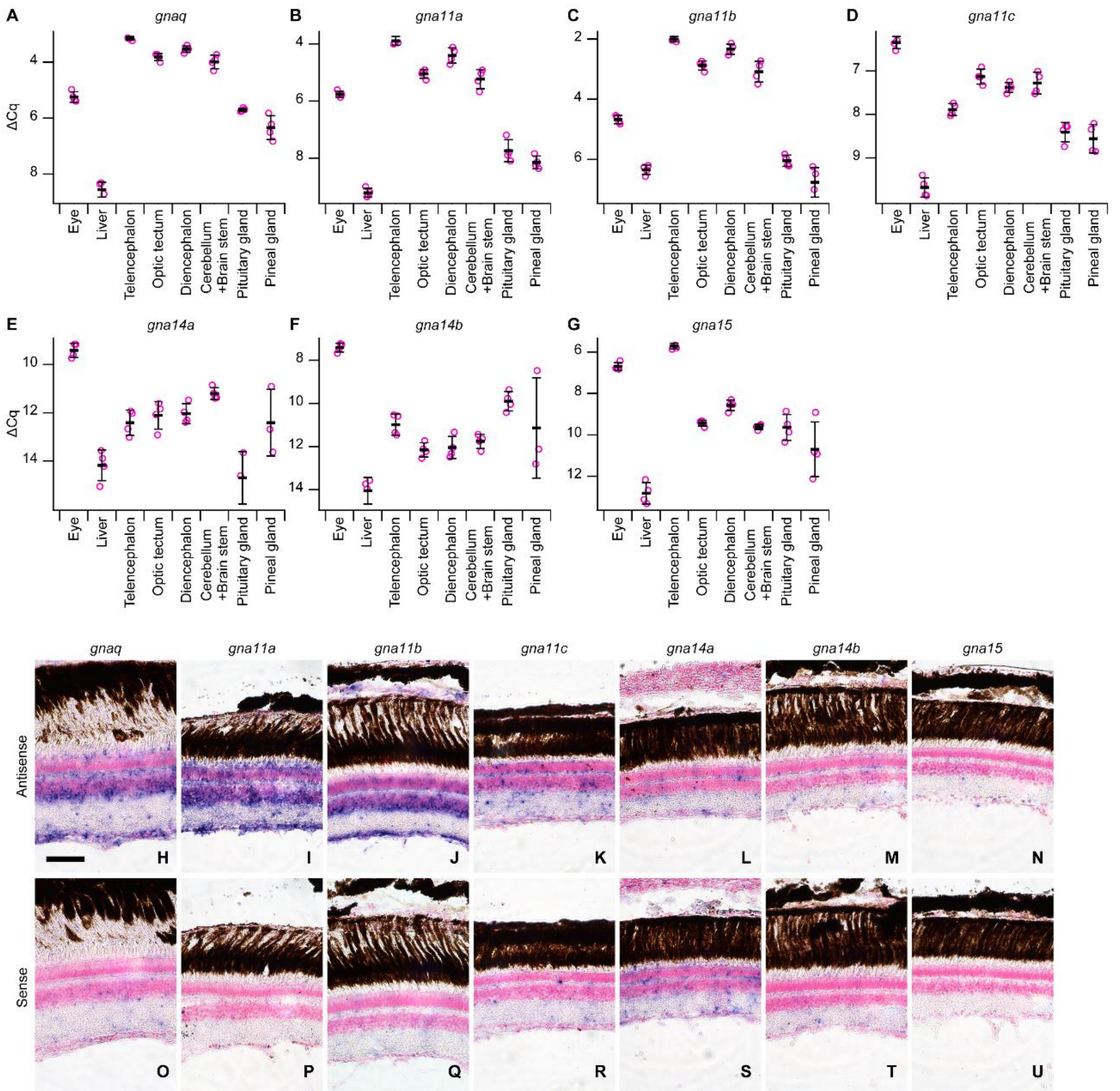

Supplementary figure 10. mRNA expression of Gq-type α subunits in the medaka eye. mRNA expression analysis of Gq-type α subunits in the medaka eye by quantitative PCR (A-G) and chromogenic in situ hybridization (H-U). (A-G) Quantitative PCR analysis of *gnaq* (A), *gna11a* (B), *gna11b* (C), *gna11c* (D), *gna14a* (E), *gna14b* (F), and *gna15* (G) performed on medaka neural tissues and liver. Ribosomal protein L7 (*rpl7*) served as an endogenous control reference gene. The data were indicated as ΔCq values (difference between Cq values of target and reference genes). Magenta circles and error bars show the individual data and s.e.m., respectively. ΔCq values of *gnaq*, *gna11a*, and *gna11b* are less than 6 in the eye, while those of the rest of *gna* genes are 6 to 10 in the eye, indicating that the latter *gna* genes are expressed at lower levels. (H-U) Chromogenic in situ hybridization of *gnaq* (H, O), *gna11a* (I, P), *gna11b* (J, Q), *gna11c* (K, R), *gna14a* (L, S), *gna14b* (M, T), and *gna15* (N, U) performed on medaka retinas. Panels H-N show retinal sections hybridized with antisense

probes. Panels O-U show the consecutive tissue sections of H-N, respectively, hybridized with corresponding sense probes as negative controls. Tissues were counterstained with nuclear fast red. Scale bar: 50 μm.

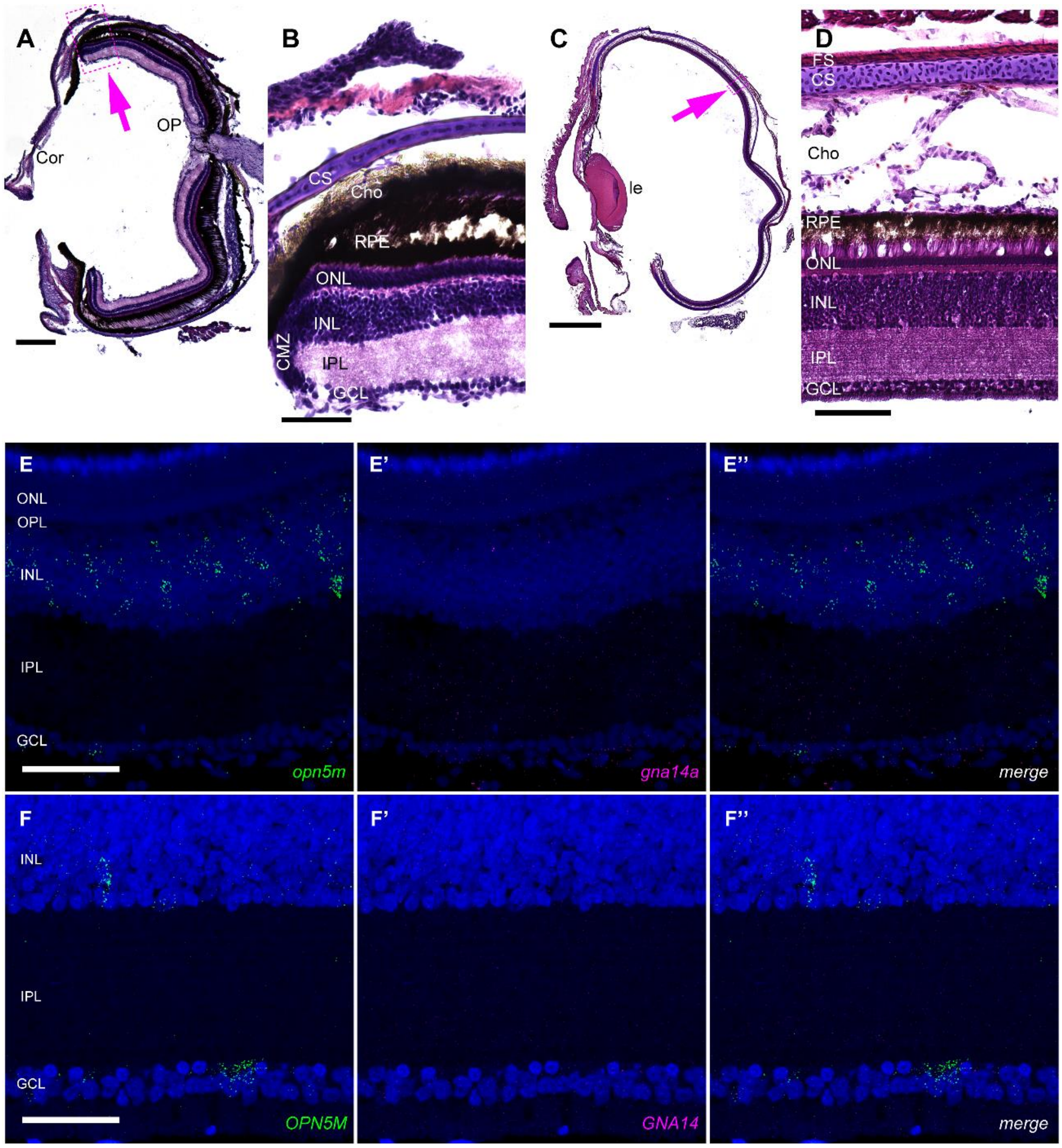

Supplementary figure 11. mRNA expression of *opn5m/OPN5M* in the medaka and chicken retina.

Histology and fluorescent in situ hybridization using signal amplification by exchange reaction (SABER). (A, B, C, D) Hematoxylin and eosin staining of medaka (A, B) and chicken (C, D) eyes. Panels B and D show the enlarged views of the region indicated by magenta dotted box and arrows in panels A and C, respectively. (E, E', F, F') Fluorescent signals of medaka *opn5m* (E), *gna14a* (E'), chicken *OPN5M* (F), and *GNA14* (F') are shown. Cell nuclei were stained with Hoechst 33342 (Blue). Panel E'' shows the merged view of panels E and E'. Panel F'' shows the merged view of panels F and F'. GCL, ganglion cell layer; Cho, choroid; CMZ, ciliary marginal zone; Cor, cornea; CS, cartilaginous sclera; FS, fibrous sclera; INL,

inner nuclear layer; IPL, inner plexiform layer; le, lens; ONL, outer nuclear layer; OP, optic papilla; OPL, outer plexiform layer; RPE, retinal pigment epithelium. Scale bar: A, 200 μm; B, E, F, 50 μm; C, 2 mm; D, 100 μm.

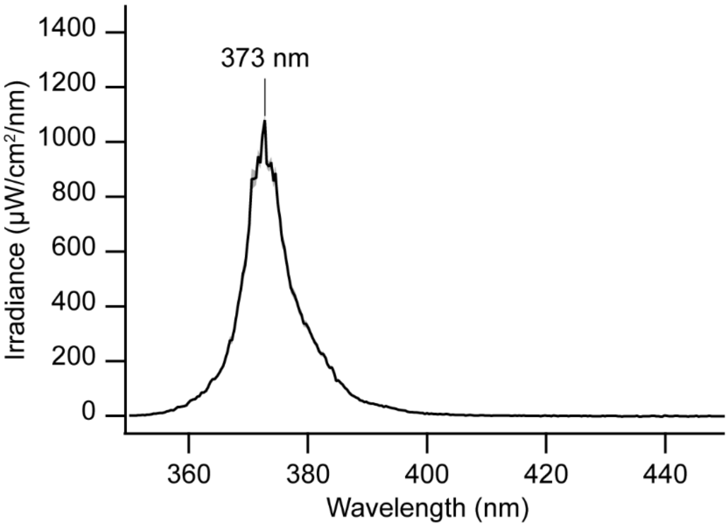

Supplementary figure 12. Irradiance spectrum and deviation of UV light source

Irradiance of a handheld UV flashlight used for stimulation of opsins in this study was measured by USB2000+ spectrophotometer (OceanOptics) connected to the CC-3 cosine corrector (OceanOptics). Distance between the light source and the cosine corrector was adjusted to 1.4 cm, which is from the bottom of the 96 well plate to the light source. Black trace and shading show mean ± standard deviation (ex. 1079.8 ± 21.3 at 373 nm) calculated by eight independent measurements.

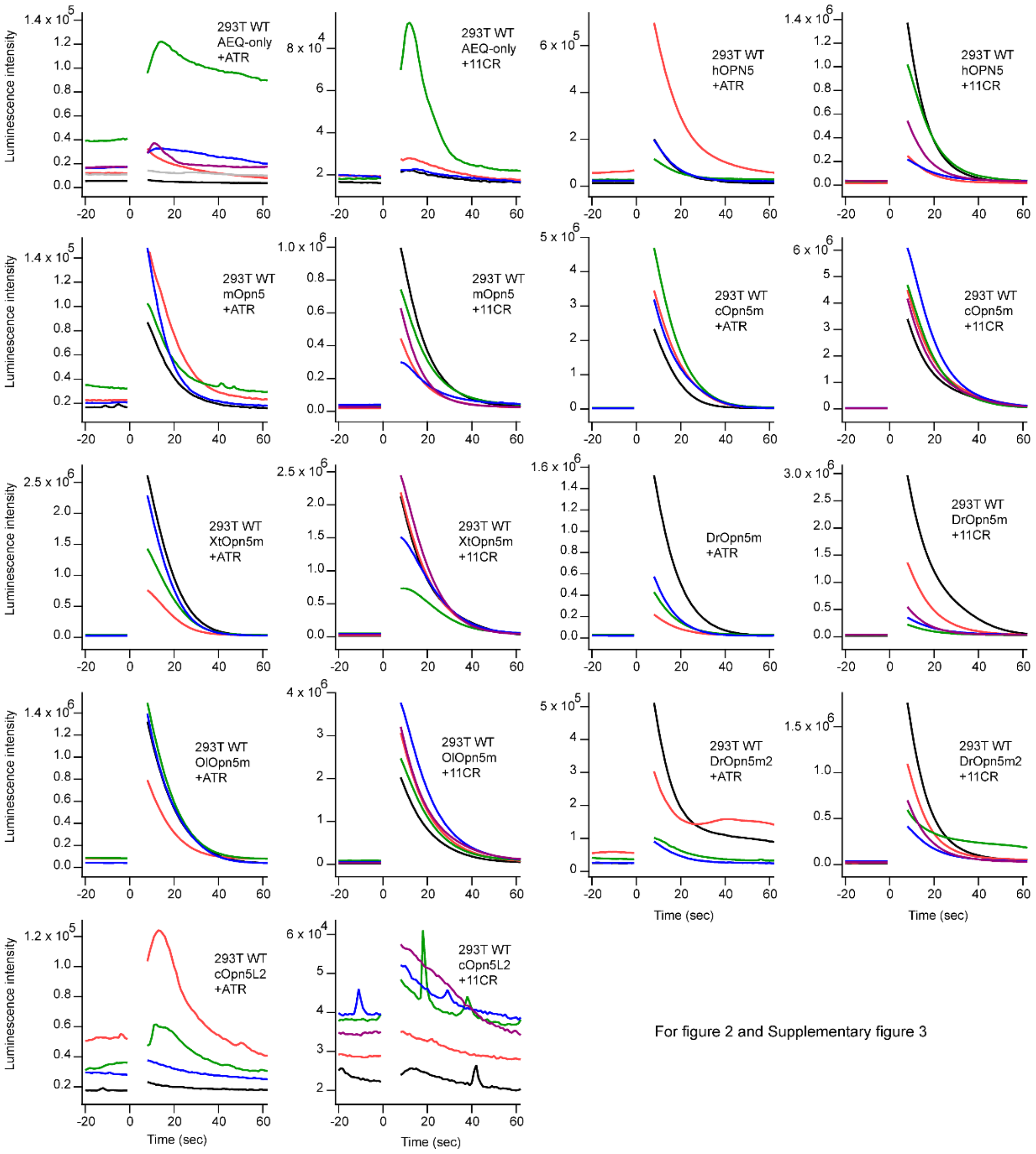

(Supplementary figure 13, continued)

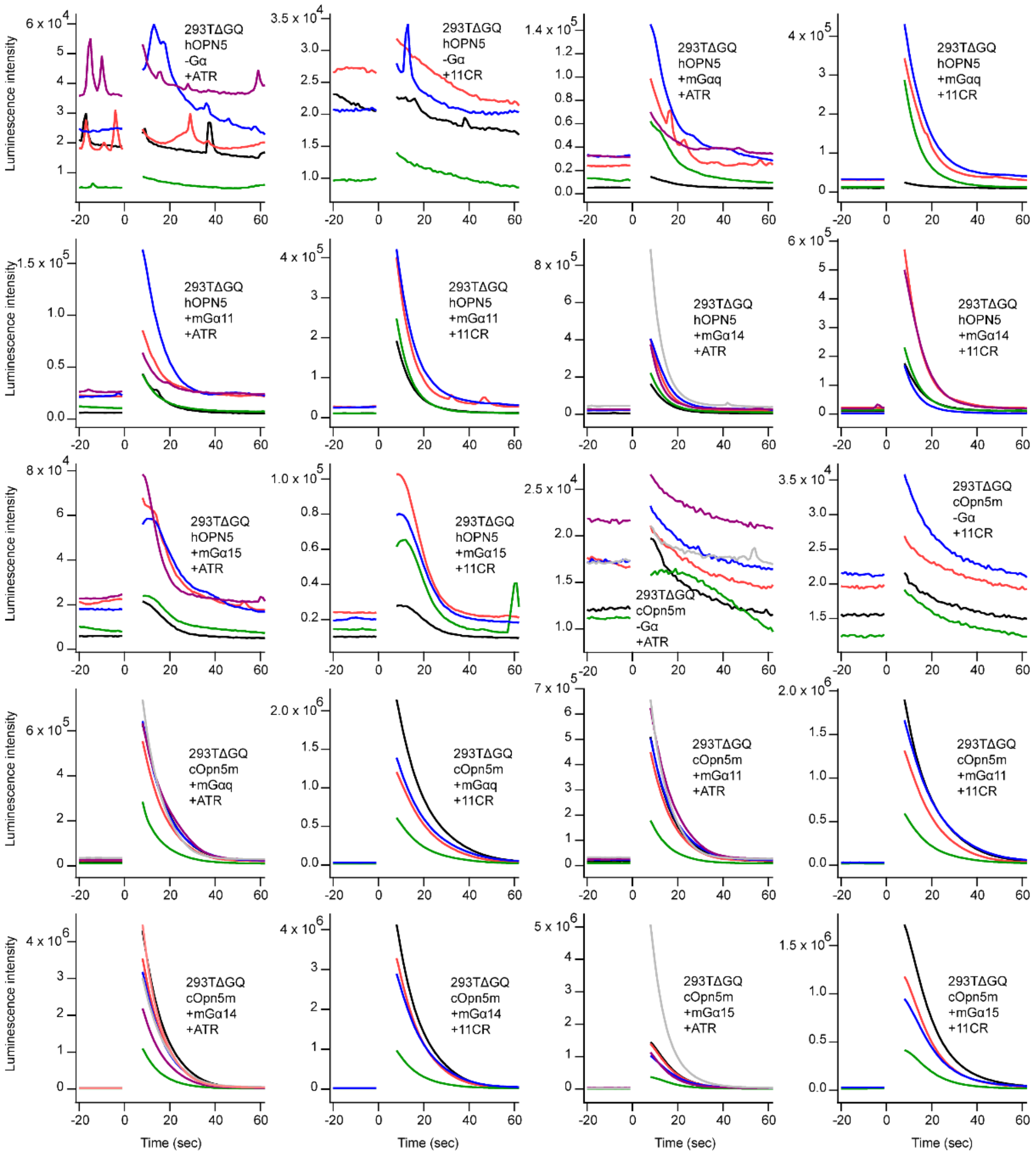

For figure 4A, B and Supplementary figure 6A, B

(Supplementary figure 13, continued)

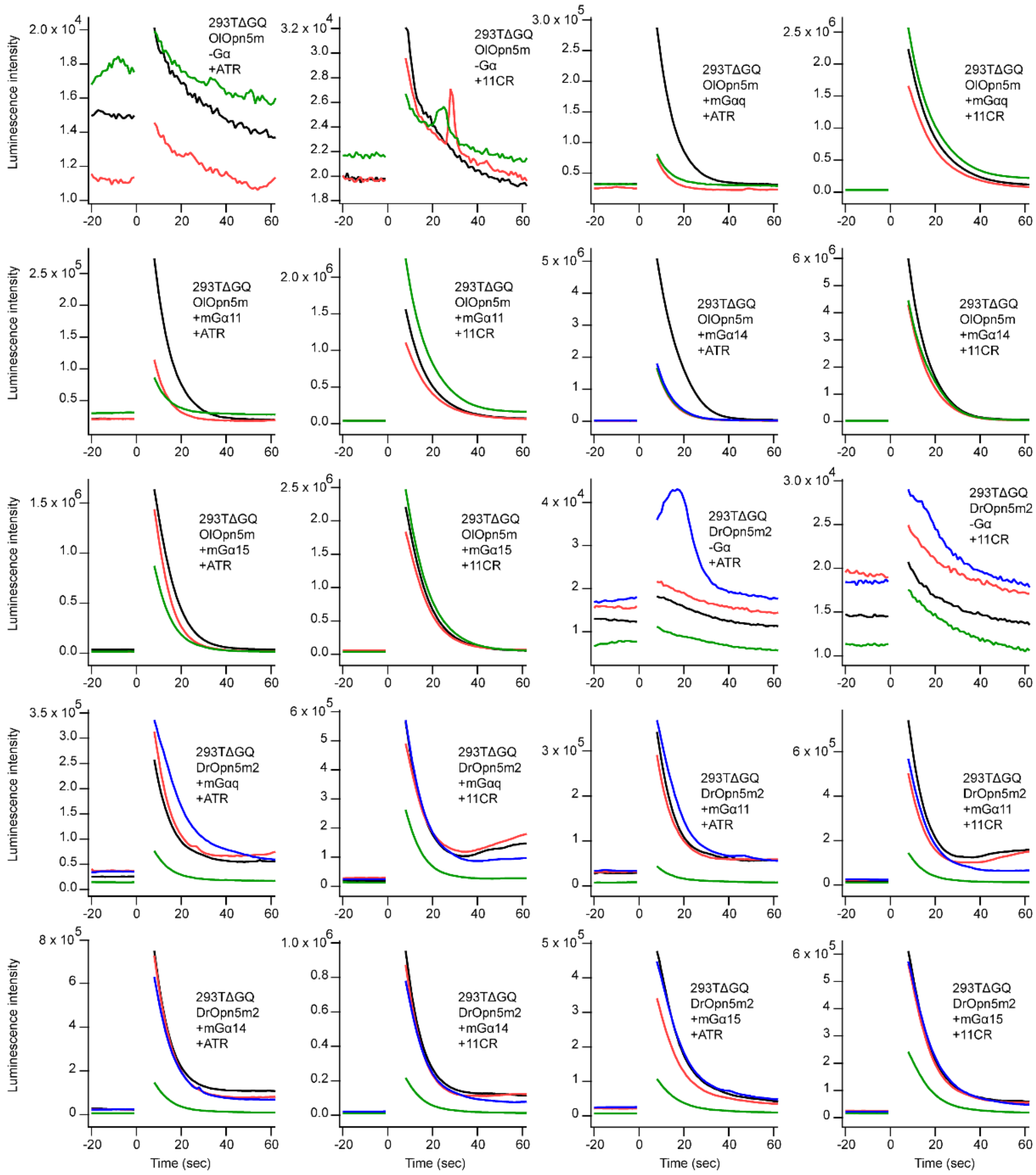

For figure 4C, D and Saupplementary figure 6C, D

(Supplementary figure 13, continued)

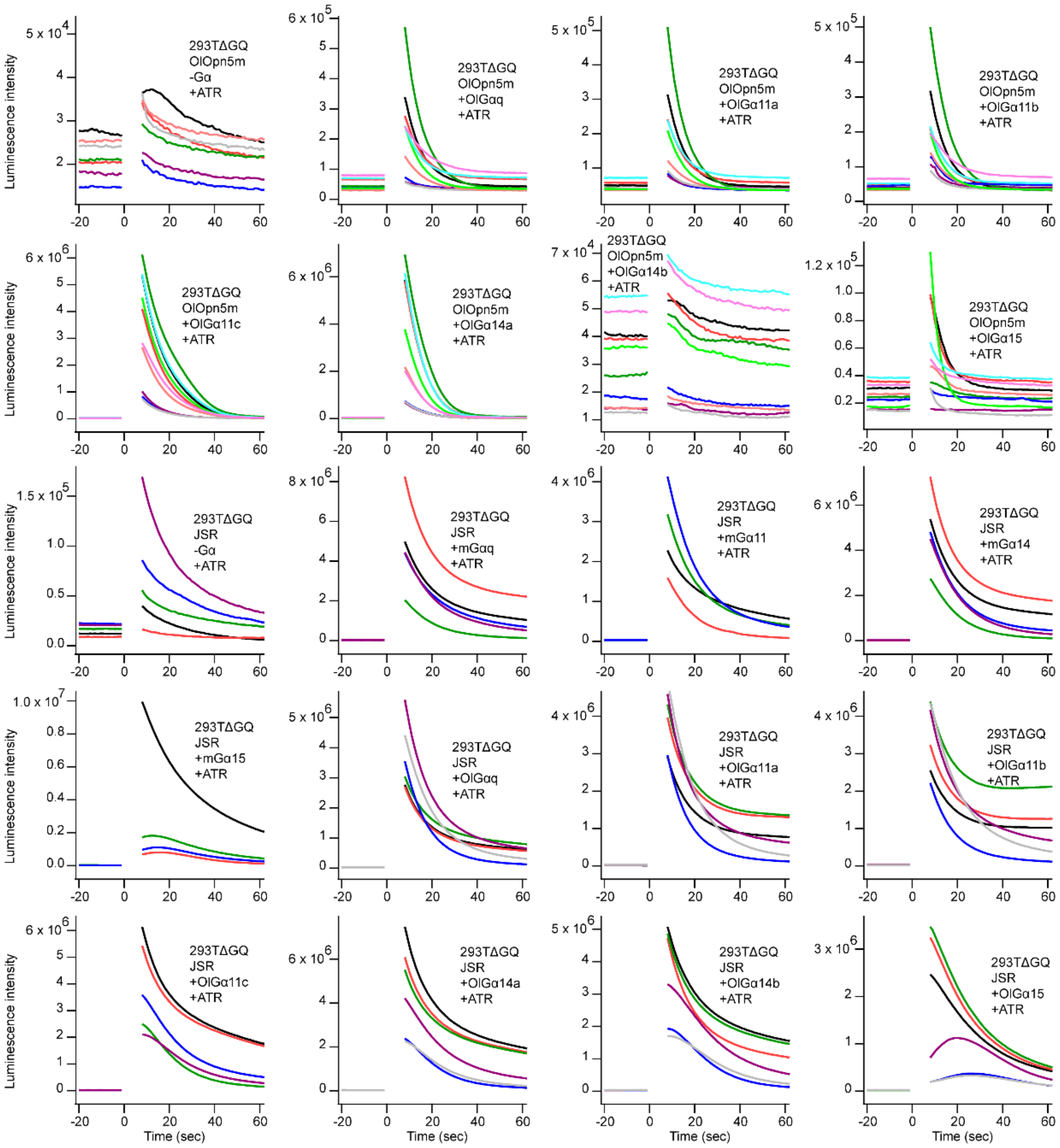

For figure 4F and Supplementary figure 8

(Supplementary figure 13, continued)

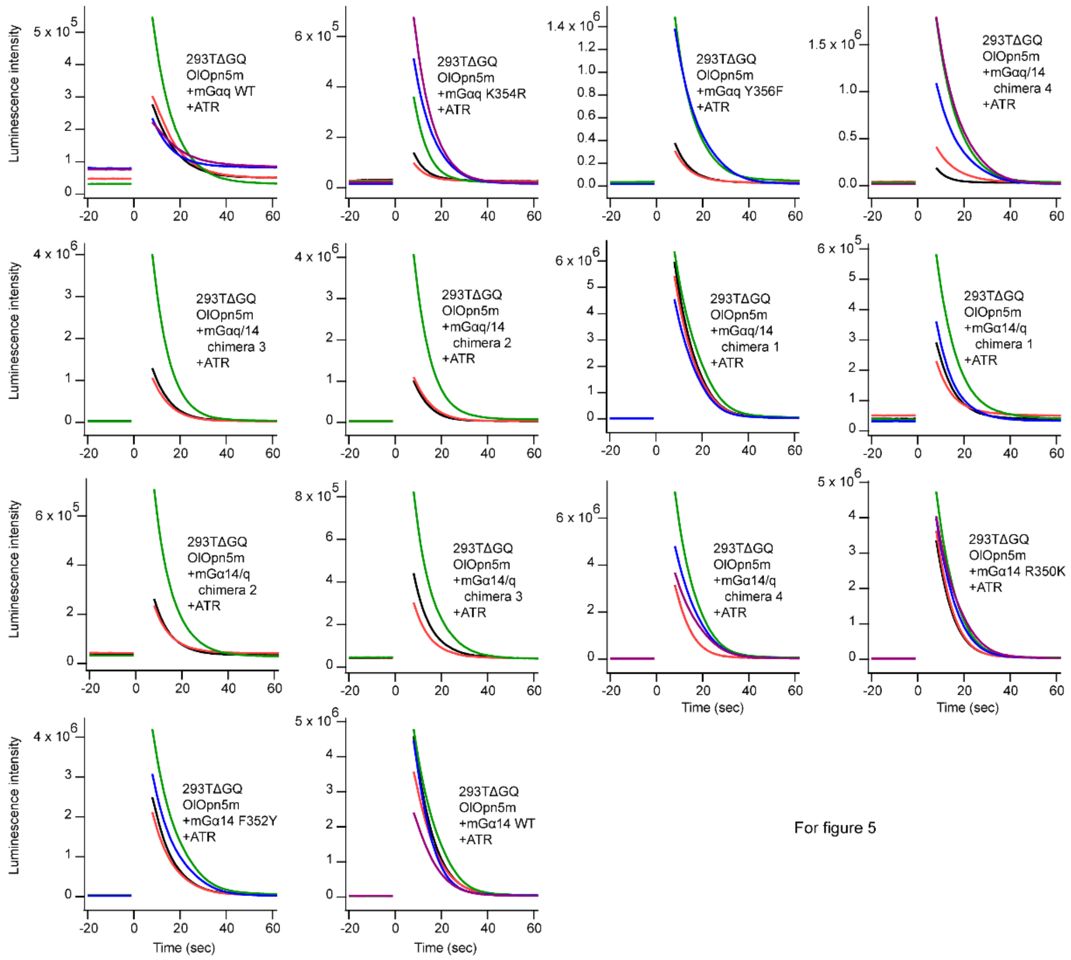

(Supplementary figure 13, continued)

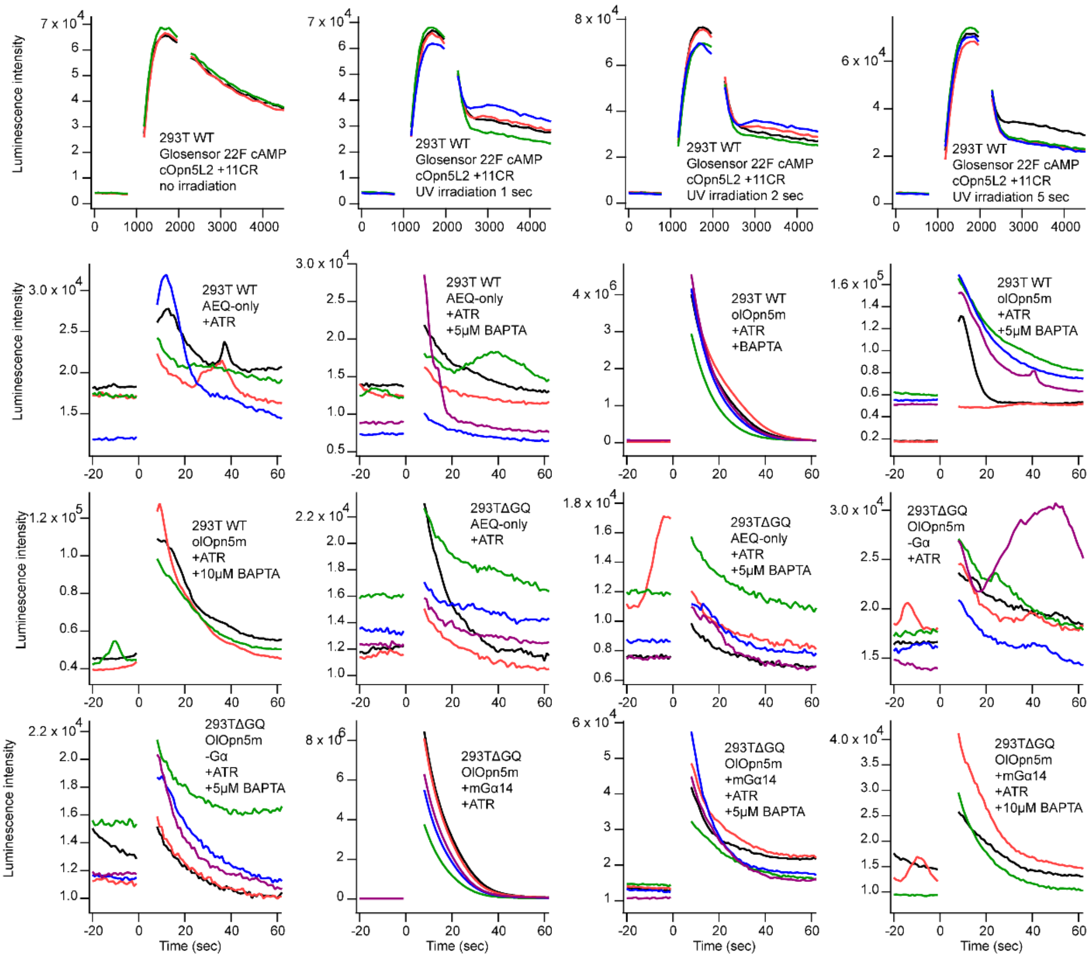

For Supplementary figure 2 and 4

Supplementary figure 13. Unmodified raw traces of luminescence measurement

Raw luminescence intensity values measured by a luminometer are shown. Light irradiation was performed at 0 sec for aequorin or at 2000 sec for Glosensor 22F. For calculation of relative luminescence, the luminescence intensity just after irradiation was divided by that immediately before irradiation. Each panel contains the data obtained in the denoted conditions (cell genotype, transfected opsin and Gα, retinal isomer, calcium chelator). Unless otherwise noted (Glosensor 22F cAMP), mitochondria-targeted aequorin N26D (AEQ) DNA was co-transfected. Each trace was differently colored just for discrimination.

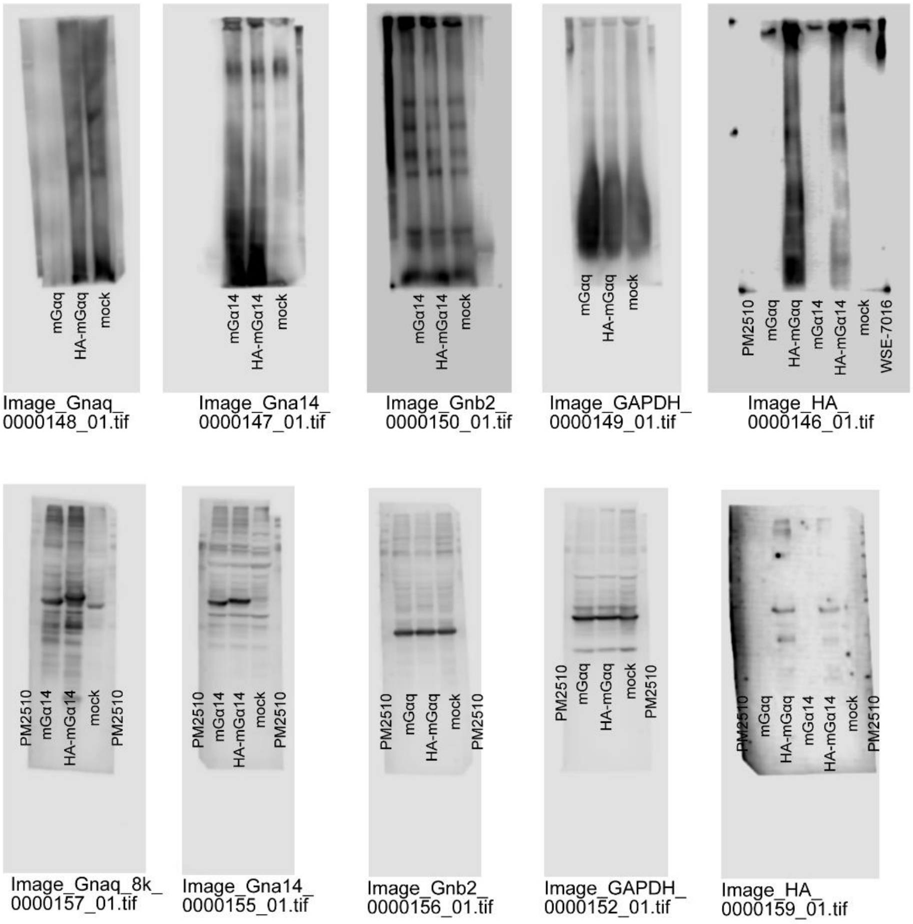

(Supplementary figure 14, continued)

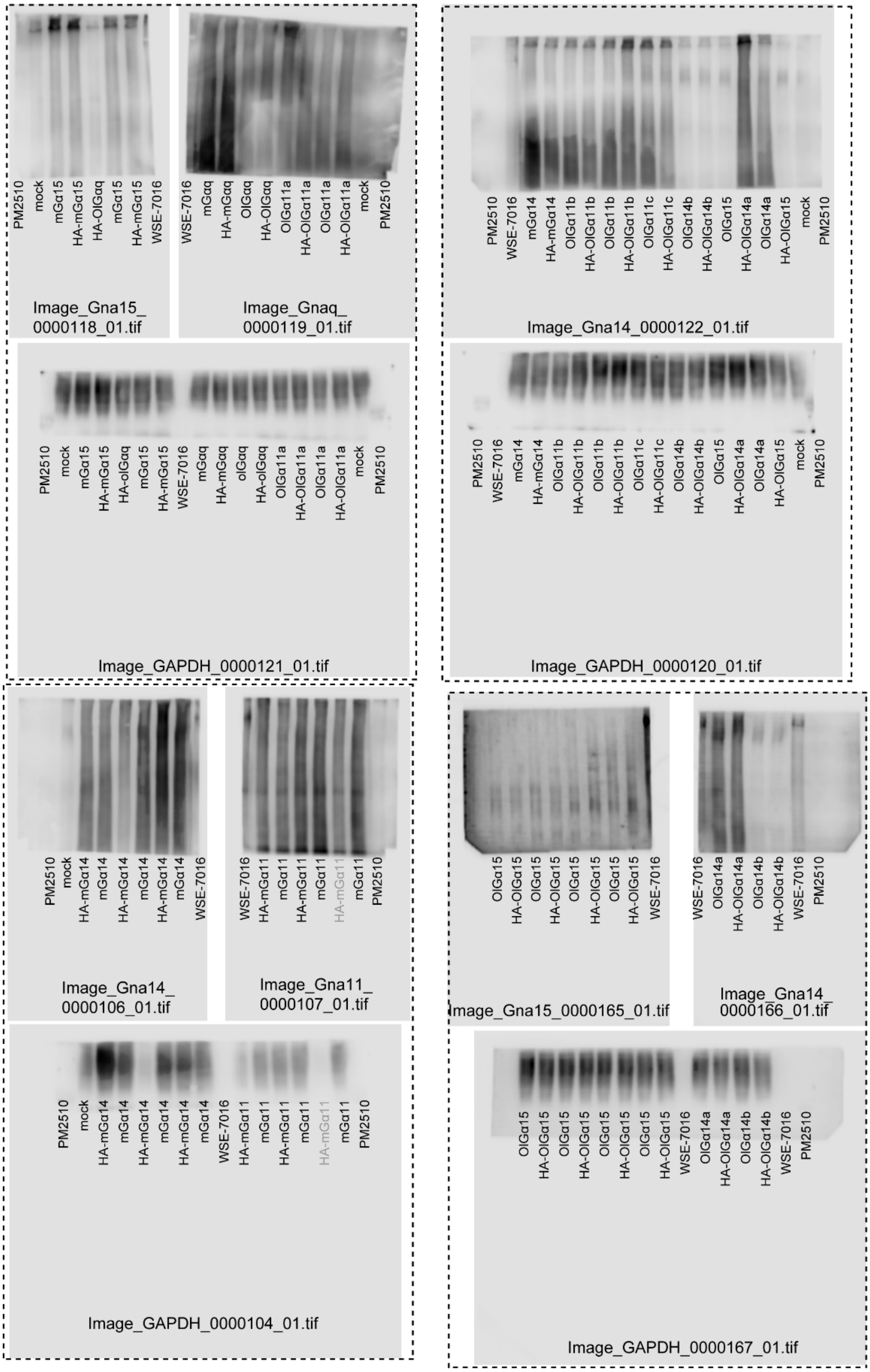

(Supplementary figure 14, continued)

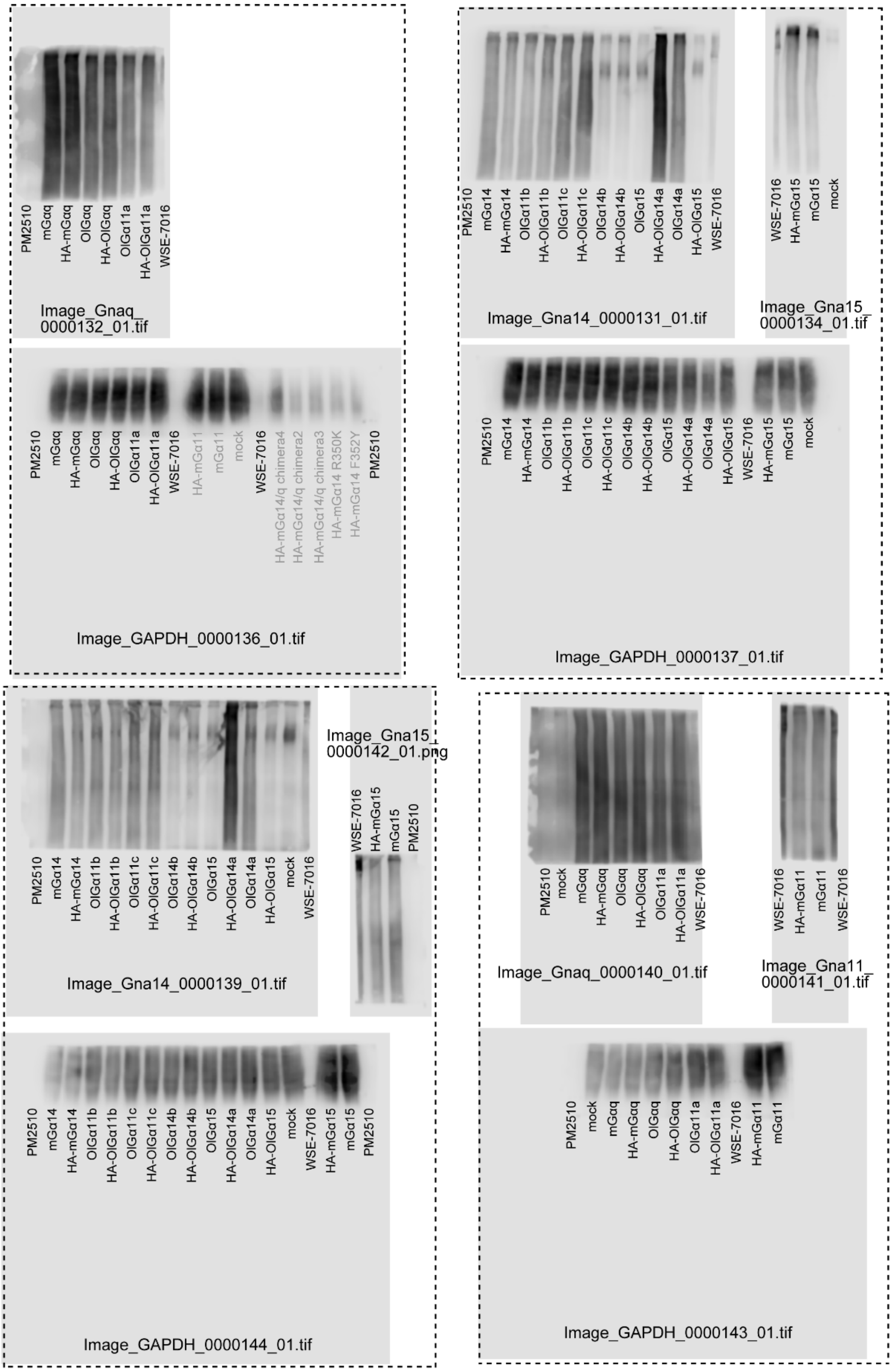

(Supplementary figure 14, continued)

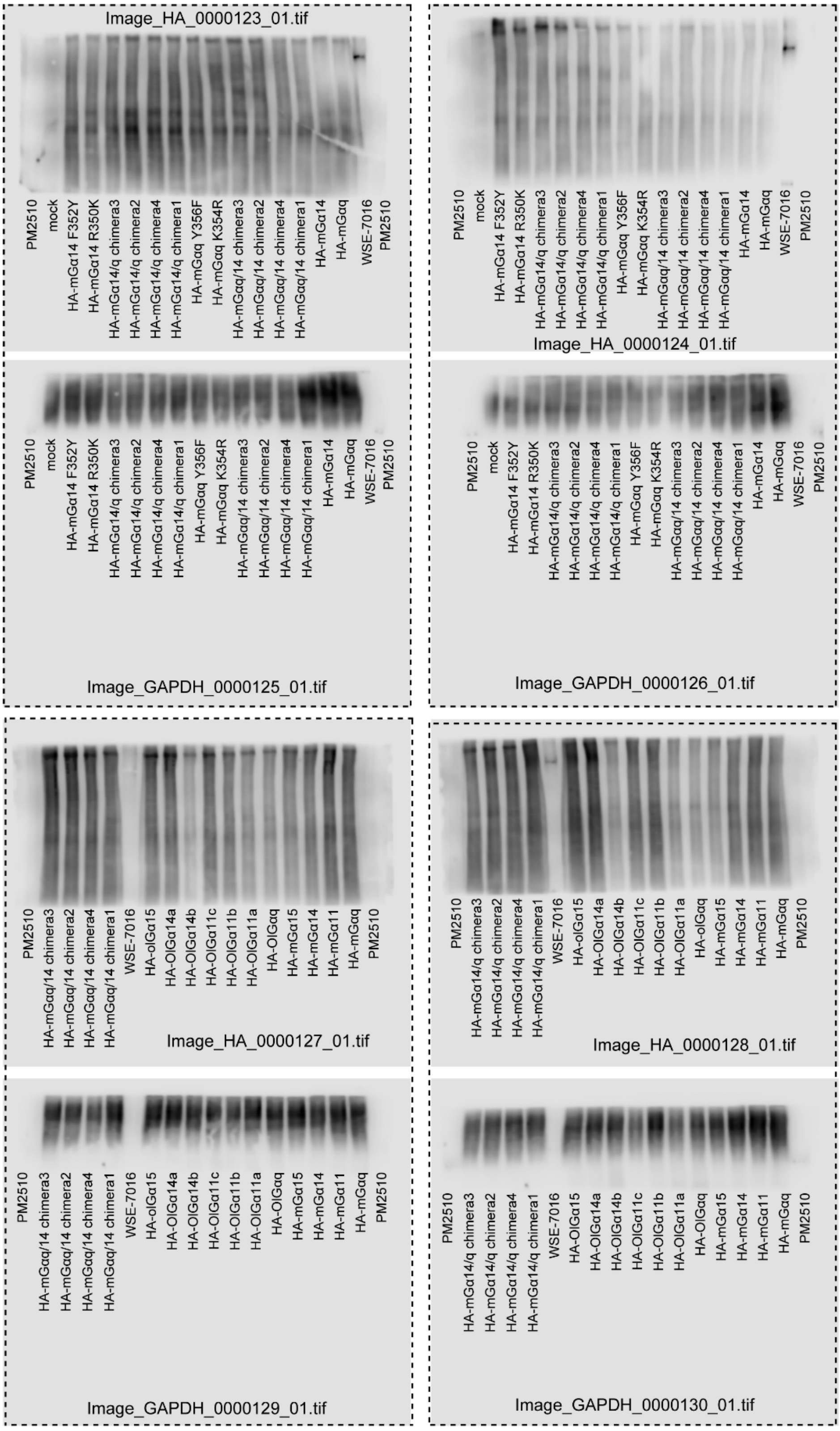

(Supplementary figure 14, continued)

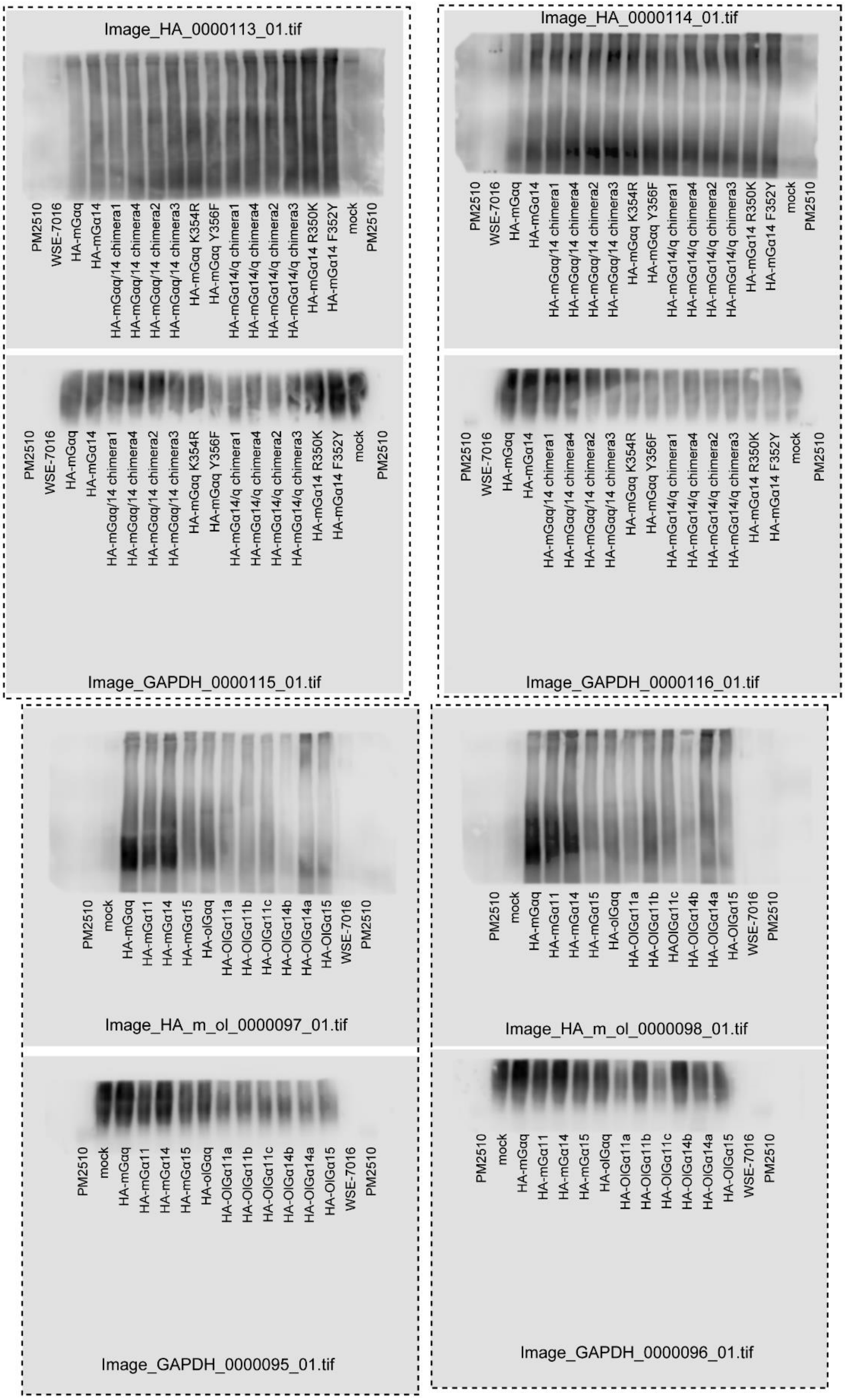

(Supplementary figure 14, continued)

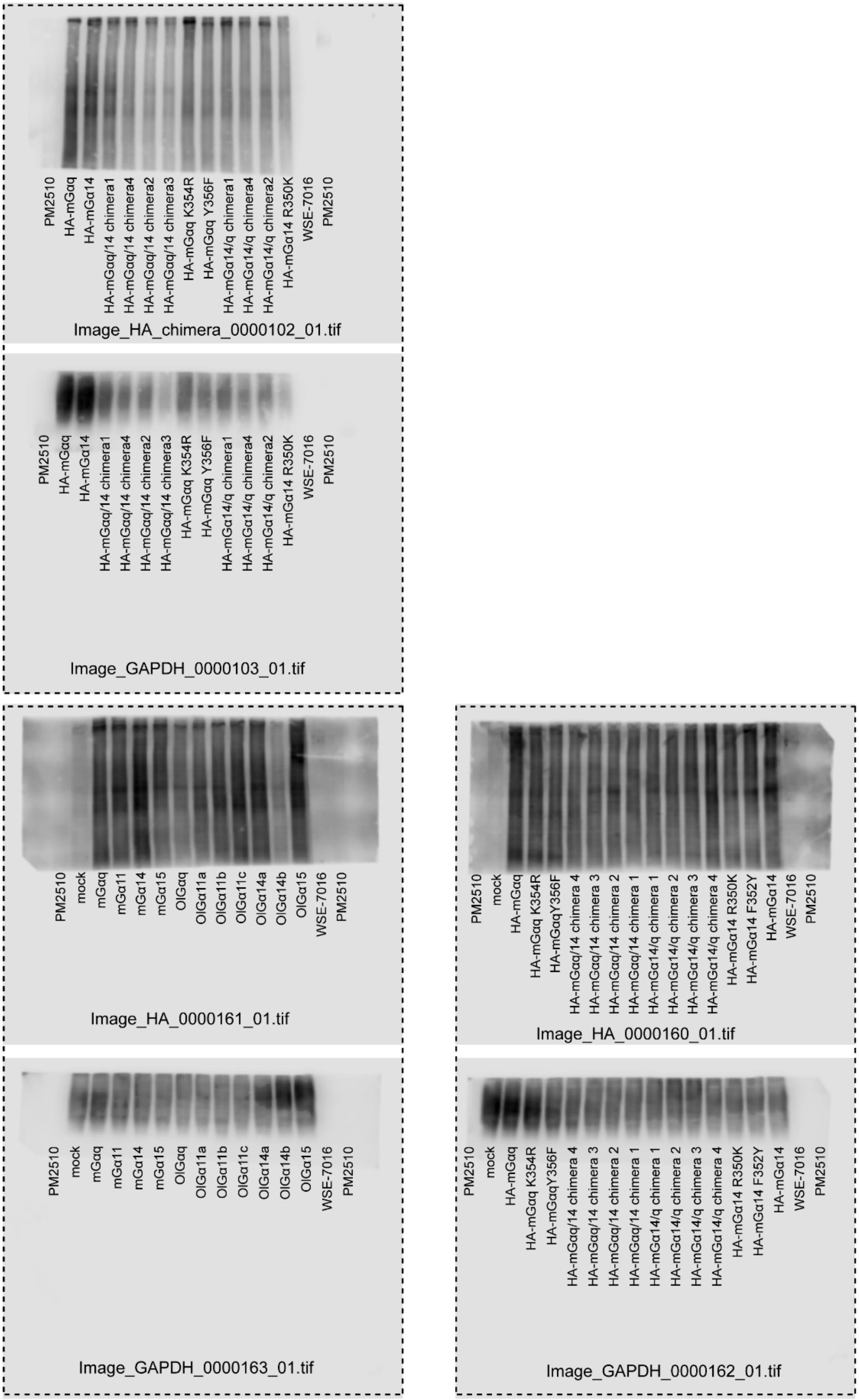

Supplementary figure 14. Uncropped images of Western blot analyzed in this study

All the uncropped images of Western blot used in this study are shown. Densitometry of bands for Supplementary figure 7 was performed based on original data using the software, Image Studio Lite Ver 5.2 and Image Studio Digits Ver 4.0 (Li-COR). Shown images were exported from the software as tiff format with denoted file names. Membranes were cut into two or more pieces for detection by other antibodies. Dotted boxes indicate that those images inside are split from the same membranes. Lanes shown in gray letters were not used for analysis because of failure in detection of GAPDH or Gα. PM2510 and WSE-7016 indicate lanes of molecular weight markers, ExcelBand enhanced 3-color regular range protein marker (PM2510, SMOBIO) and EzStandard Native (WSE-7016, ATTO), respectively.

Supplementary references

1. Sakai, K., Shichida, Y., Imamoto, Y., and Yamashita, T. (2022) Creation of photocyclic vertebrate rhodopsin by single amino acid substitution. *eLife*. **11**, e75979
2. Sato, K., Khine Nwe Nwe, and Ohuchi, H. (2021) The Opsin 3/Teleost multiple tissue opsin system: mRNA localization in the retina and brain of medaka (Oryzias latipes). *J. Comp. Neurol*. **529**, 2484–2516
3. Katoh, K., and Standley, D. M. (2013) MAFFT multiple sequence alignment software version 7: improvements in performance and usability. *Mol. Biol. Evol.* **30**, 772–780
4. Drastichova, Z., and Novotny, J. (2012) Identification of a Preassembled TRH Receptor-$G_{q/11}$ Protein Complex in HEK293 Cells. *Cell Struct. Funct.* **37**, 1–12
5. Drastichova, Z., and Novotny, J. (2012) Identification and subcellular localization of molecular complexes of Gq/11α protein in HEK293 cells. *Acta Biochim. Biophys. Sin. (Shanghai).* **44**, 641–649
6. Wilson, P. T., and Bourne, H. R. (1995) Fatty acylation of alpha z. Effects of palmitoylation and myristoylation on alpha z signaling. *J. Biol. Chem*. **270**, 9667–9675
